# Reliable Single-Trial Detection of Saccade-Related Lambda Responses with Independent Component Analysis

**DOI:** 10.1523/ENEURO.0270-25.2025

**Published:** 2025-11-21

**Authors:** Iffah Syafiqah binti Suhaili, Balint Toth, Zoltan Nagy, Zoltan Juhasz

**Affiliations:** University of Pannonia, Veszprem 8200, Hungary

**Keywords:** EEG, ICA, lambda wave, saccade, single-trial, visual perception

## Abstract

In natural, free-viewing settings, visual perception is driven by a series of saccades and fixations. Perceptual mechanisms are typically studied through averaged fixation-related potentials generated from simultaneous eye-tracking and EEG recordings. Lambda responses following fixation onsets signal the arrival of new visual input to the primary visual cortex. In our study, we investigate the use and preprocessing parameter dependence of independent component analysis (ICA) in separating the lambda response from other neural sources. In our experiment, 10 subjects (2 males and 8 females) viewed 80 art paintings in natural, free-viewing settings, during which EEG data were recorded. Our results show that unique lambda response components can be detected reliably and individual lambda waves can be extracted in a single-trial manner, without signal averaging. ICA decomposition is most sensitive to high-pass filtering producing best results with a minimum 1 Hz filtering. We also propose a method that automatically and accurately identifies the lambda component among other independent components for further lambda peak detection. These individual lambda waves can then be used to study saccade-related modulation effects without losing temporal and spatial resolution. The novelty of our method is the automatic detection of lambda components and extraction lambda waves, which is a new approach in saccade/fixation and visual perception research under naturalistic viewing conditions.

## Significance Statement

Understanding saccade-related visual processes in naturalistic viewing conditions is a key challenge in visual perception research. The present study demonstrates for the first time that by using suitable parameter settings, Independent Component Analysis can reliably detect and extract lambda waves from continuous EEG signals in a single-trial manner. Our proposed method detects individual lambda components automatically and then extracts lambda waves with or without eye-tracking information in a single-trial manner, which is suitable for studying individual saccade–lambda response pairs without signal averaging. We show that the method is robust under a wide range of processing parameters. This work highlights the potential of using single-trial lambda analysis as a new tool to advance our understanding of visual perception and cognition.

## Introduction

Most cognitive EEG experiments take place under strictly controlled laboratory conditions, where participants must sit still in front of a computer display and respond to short, repeated stimuli while instructed to avoid eye movements. In several experimental scenarios, these rather unrealistic conditions cannot be guaranteed, e.g., studying brain activity during continuous sentence reading, natural scene exploration, vehicle driving, or museum visits. In such free-viewing tasks, the simultaneous use of eye-trackers can help identify saccades and fixations and provide EEG researchers with the ability to extract saccade or fixation-related potentials (FRPs). Since saccades are dominant artifacts in free-viewing EEG experiments, significant effort has been devoted to the identification and removal of saccade-related artifacts (Mennes et al., 2010; Craddock et al., 2016; Dimigen, 2020; Madison et al., 2025). Independent component analysis (ICA), a form of blind source separation (BSS) method, is routinely used to separate mixed neural and non-neural sources based on the assumption of statistical independence, non-Gaussian distribution, and linear mixing of sources (Jung et al., 1998). ICA has been shown to reliably identify ocular, muscle, heart-related artifacts that, in turn, can be removed from the measured signals.

In free-viewing experiments, however, eye movements are not simply nuisance factors but carry additional important information about the execution and timing of tasks, level of attention or engagement. Strongly related to saccades are lambda responses that manifest themselves as sharp, sawtooth-like positive-going peaks (lambda waves) following saccade offsets (equivalent to fixation onsets) that represent the response to the new visual input reaching the primary visual cortex after the saccades (Evans, 1953). The lambda response has been studied extensively (Evans, 1953; Yagi, 1979; Billings, 1989; Ries et al., 2018; Madison et al., 2025) to understand its physiological mechanism and dependence on bottom-up perceptual parameters, using the traditional EEG/ERP methodology relying on ensemble averaging of many repeated trials. With averaging epochs, one can create saccade onset or offset-related potentials. Yagi (1979) demonstrated that the lambda response (a component of saccade-related potentials) is evoked by saccade offset rather than onset. Since the saccade offset coincides with the fixation onset, some authors refer to the saccade offset-related potential as FRP (Dimigen et al., 2011; Touryan et al., 2017; Ries et al., 2018). While epoch averaging improves signal-to-noise ratio, it also blurs the signal in the time domain, reducing its temporal resolution. If multiple electrodes are also averaged, mixing effects and reduction in spatial resolution can be expected.

Despite the importance of the lambda response, its detection received less attention, especially in free-viewing experiments. Since ICA can unmix neural sources as well, our hypothesis is that if lambda responses are localized to a distinct occipital cortical region and manifest themselves as a characteristic signal pattern, ICA should be able to extract lambda responses as an independent component, which would allow us to perform single-trial analysis based on individual lambda waves.

The goal of this work is to investigate the suitability of ICA for lambda wave detection and extraction in a single-trial manner using EEG data from a free-viewing neuroaesthetic experiment, without relying on signal averaging, and to study the reliability of the method under different preprocessing parameter settings. If we can extract individual lambda waves and analyze them by correlating their amplitude, width, and latency with either saccade (amplitude, duration, velocity) or low- and high-level image parameters (change in color, contrast, texture, luminance, content, style, and context), we may be able to uncover perceptual processes more accurately. In addition, extracting unmixed cortical source activations may help in understanding what areas are involved in processing the incoming information flow, in what order, and how these sources interact following each saccade.

## Materials and Methods

### Data acquisition

#### Participants

Ten right-handed university students (2 males and 8 females, mean age = 23.4 years, SD = 4.7) volunteered to participate in this study. Each participant had normal or corrected vision and had no history of neurological or psychiatric disorders. The participant's lateral dominance was determined using the Edinburgh Handedness Inventory (Oldfield, 1971) and to exclude potential confounding handedness effects. Participants were briefed about the nature of the study, the risks and benefits, and their right to withdraw at any time. Written informed consent was obtained from every participant confirming their participation and permission for the data to be used for research and publication purposes. The experiment was approved by the Ethical Committee of the Authors’ University. Data from one participant who had excessive eyeblinks during the experiment was excluded from the study; hence, data of the remaining nine participants (two males and seven females) are analyzed and presented here.

#### Stimuli

We presented 80 color art paintings in a random sequence to each participant on a 21″ computer display (screen size, 43.5 × 27 cm; resolution, 1,920 × 1,080 pixels; refresh rate, 60 Hz) from an average viewing distance of 72.1 cm (SD = 2.06). Paintings included a set of representational and abstract artworks with content spanning portrait, landscape, townscape, still life, groups of people, and various geometrical forms and textures. To promote a natural viewing experience, no chin rest was used during the experiment; participants were instructed to explore the paintings freely as if they were visiting an art gallery but with minimal head and body movement.

#### Procedure

**Figure 1.**
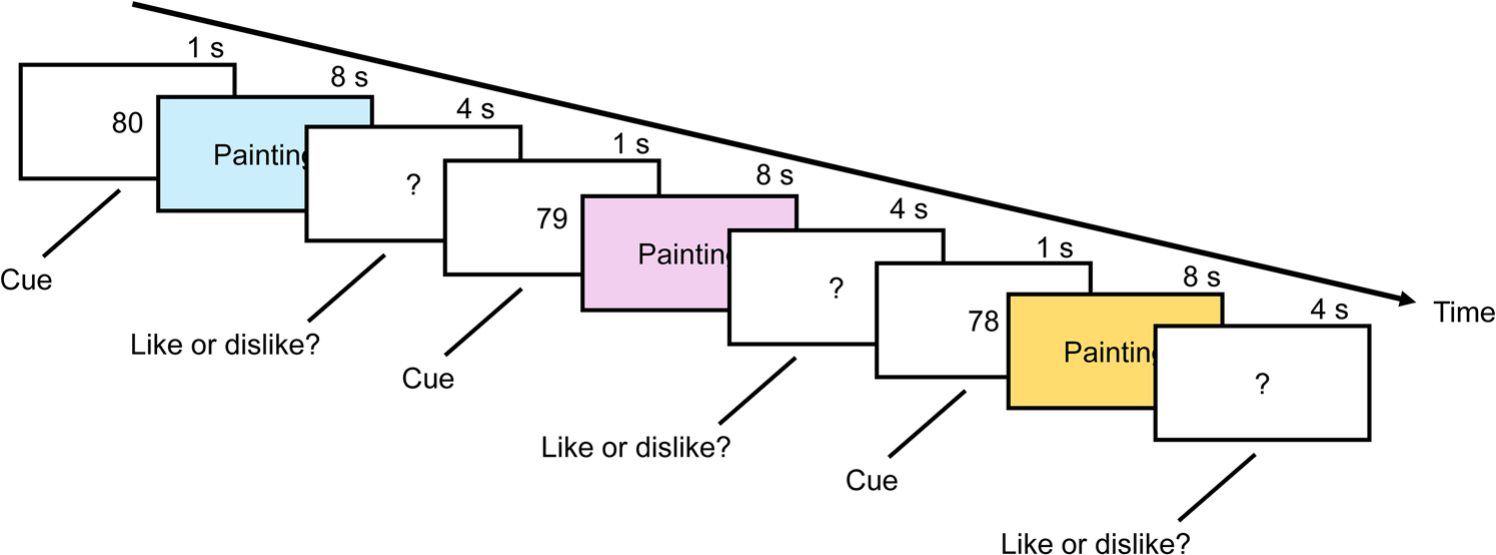
Experimental design.

The design of the experiment is shown in Figure 1. Each painting was shown for 8 s preceded by a 1 s numeric cue. The cue was the number of remaining paintings and it was used to maintain participant alertness and interest. Each painting presentation was followed by a 4 s (black screen) interval during which the participants had to respond by pressing buttons indicating either a Like or Dislike response. Response data are not analyzed and reported in this paper. The total experiment length was 1,040 s (∼18 min).

#### Recording apparatus

EEG and eye movement data were registered simultaneously during the experiment. EEG was recorded with a 128-channel Biosemi (BioSemi) ActiveTwo EEG device using Ag/AgCl active electrodes. The electrode caps used the 128-channel Biosemi ABC layout (Extended Data Fig. 4-1). Six external flat electrodes were used to record horizontal and vertical eye movements and single-lead electrocardiogram data, placed at the outer canthi, above and below the left eye and the two wrists, respectively. DC electrode offset values were kept stable and below the recommended 40 μV amplitude level. The analog EEG signal was low-pass filtered (fc = 410 Hz, −3 dB) internally by the EEG device before being digitized at a sampling rate of 2,048 Hz. Data were saved in BDF EEG file format for later offline processing. Eye movement was recorded with a Tobii (Tobii) Pro Fusion screen-based eye-tracker device at a sampling rate of 250 Hz. The accuracy and precision of the eye-tracker are 0.3° and 0.2°, respectively. The Tobii Pro Lab software (v24.21.435 x64) was used for both stimuli presentation and eye movement data registration. Since the eye-tracker device does not provide external device synchronization capability, a white square of size 1 cm^2^ was displayed along with each painting for 8 s at the right edge of the screen. This was then detected by an on-screen photo-sensor that generated trigger signals for the EEG device on the appearance of the white square. The trigger event marked the start of each painting in the EEG recordings and enabled offline synchronization of the eye-tracking and EEG datasets.

### Eye movement data

**Figure 2.**
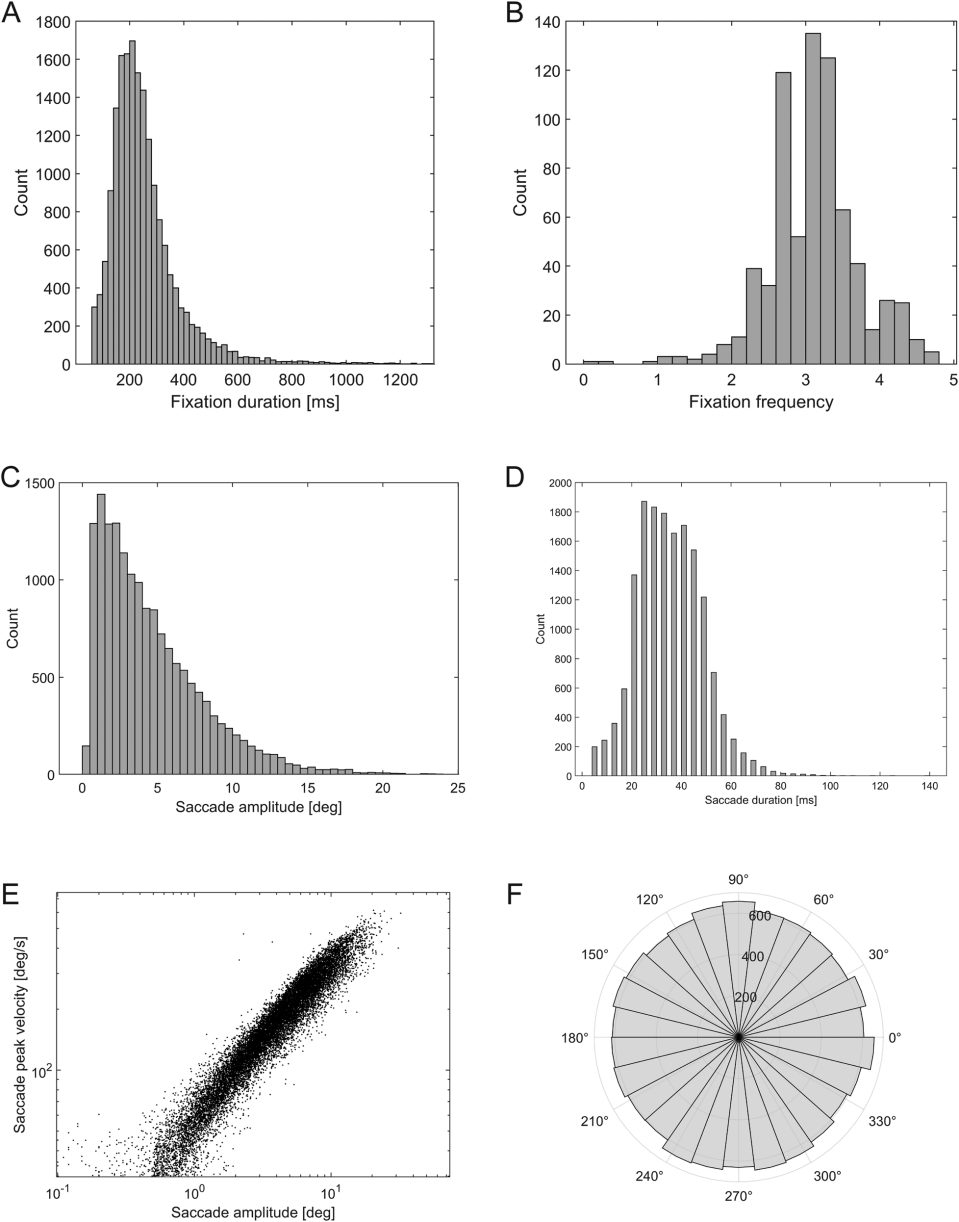
Eye movement data combined from all participants. ***A***, Distribution of the fixation duration. ***B***, Distribution of the fixation frequency. ***C***, Distribution of the saccade amplitude. ***D***, Distribution of the saccade duration. ***E***, Saccade main sequence. ***F***, Saccade angular histogram.

**Figure 3.**
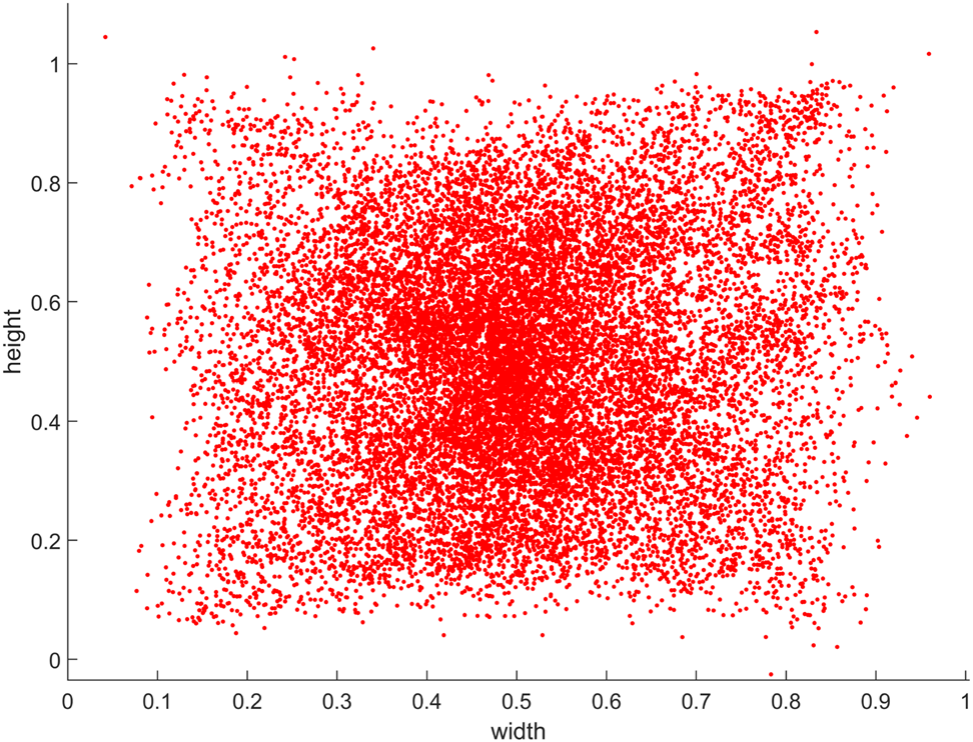
Distribution of the normalized fixation points of all subjects and paintings.

For each participant, saccade and fixation events were extracted from the measurements using the Tobii Pro Lab software package. The Tobii software uses the I-VT velocity threshold-based fixation detection algorithm (Olsen, 2012). Saccade and fixation extraction was performed by the software using its default detection parameters (velocity threshold, 30°/s; window length, 20 ms; max. gap length for gap filling, 75 ms; min. fixation duration, 30 ms). Only the 8 s painting presentation sections were used for saccade and fixation extraction; the numeric cue and blank response intervals were ignored. The mean fixation frequency was 3.10 s^−1^ (SD = 0.6142), while the mean saccade frequency was 2.81 s^−1^ (SD = 0.6050). The summary group statistics are shown in Figure 2. Due to the nature of the task, viewing paintings containing many small visual details, saccades tended to be small (mean = 4.61°, median = 3.73°, SD = 3.498°). The saccades angular histogram (Fig. 2*F*) shows a uniform distribution due partly to the varying painting aspect ratios (i.e., different portrait and landscape orientations) and partly to the fact that participants scanned all parts of the paintings in a rather uniform manner (Fig. 3). The saccade main sequence (Fig. 2*E*) shows that most saccades fell into the amplitude range of 1–10 visual degrees but saccades were also detected within the microsaccade range (<1 degree).

The initial analysis of the eye-tracking data confirmed that participants had produced saccades and fixations in this experiment; consequently, we can expect the presence of lambda responses, whose detection by ICA is the goal of this study.

### EEG processing pipeline

EEG data were processed by custom scripts using the EEGLAB v2023.1 software environment (Delorme and Makeig, 2004) running on MATLAB v2023b (The MathWorks). The processing pipeline consisted of the key steps routinely used in EEG preprocessing (Delorme and Makeig, 2004; Bigdely-Shamlo et al., 2015; Gabard-Durnam et al., 2018): changing sampling rate, a FIR high-pass filter, a FIR low-pass filter, bad channel detection and interpolation, ICA based on the Extended Infomax ICA (Bell and Sejnowski, 1995) algorithm, and independent component classification using the ICLabel (Pion-Tonachini et al., 2019) algorithm. In the rest of the paper, we refer to this processing pipeline as *pipeline* (fs, HP, LP), where sampling rate fs, high-pass HP and low-pass LP filter cutoff frequencies are input parameters to the pipeline. The exact parameter settings and further details of the pipeline stages are described later in relevant sections. Note that the pipeline was executed on continuous EEG data. As the final step, the EEG data were segmented into 13 s epochs spanning −2 to 11 s aligned to stimulus (painting) presentation onset.

Due to the small number of participants, bad channel detection and interpolation was performed manually after visual inspection. For three participants, we detected bad electrodes (excessive noise, unstable contact) during measurement that were noted and later verified by inspecting the registered datasets. These channels (8 electrodes for subject S2, 1 for S6, and 2 for S7) were then interpolated in EEGLAB using the spherical spline interpolation method (Perrin et al., 1989) with the pop_interp() function.

ICA is used in EEG processing to identify and remove artifacts. Due to the well-known EEG volume conduction effect (Nunez and Srinivasan, 2005), EEG scalp signals are mixtures of neural and non-neural sources. ICA performs BSS (Cong, 2019) based on the assumptions that the number of sources are the same as the number of sensors, the sources are statistically independent, the distribution of source signals is non-Gaussian, and the mixing is instantaneous, i.e., no signal propagation delays are present (Makeig and Onton, 2011). Mathematically, the measured scalp EEG is given as x=As, where x is the vector of scalp electrode potentials, s represents the true sources, and A is the mixing matrix. ICA solves this equation s=Wx for s (the true sources, a.k.a the independent components) by estimating the mixing matrix A than taking its inverse $W=A^{-1}$.

Based on the assumptions that eye-, heart-, and muscle-related artifact sources as well as line noise are statistically independent from neural sources, artifacts can be separated from neural sources. While artifacts present serious problems in EEG processing, in this paper we are not concerned by the detection and removal of artifacts. Our goal is to investigate the performance of ICA to identify unique neural sources and particularly investigate whether the lambda response can be detected as a single independent component.

### Exploratory lambda component detection

The ability of ICA to detect a lambda response component was first tested by executing an EEG pipeline on the EEG datasets using commonly used settings, *pipeline* (fs = 512, HP = 1 Hz, LP = 40 Hz). Downsampling to 512 Hz sampling rate was used to speed up data processing. Performing high-pass filtering at 1 Hz has been shown to be beneficial to obtaining good ICA performance (Groppe et al., 2009; Bigdely-Shamlo et al., 2015; Winkler et al., 2015; Pedroni et al., 2019; Klug and Gramann, 2020). Low-pass filter cutoff frequencies were used in practice range from 40 to 200 Hz. For the exploratory phase, we selected 40 Hz to reduce the effects of high-frequency muscle and environmental noise and to eliminate potential line noise (50/60 Hz) effects.

**Figure 4.**
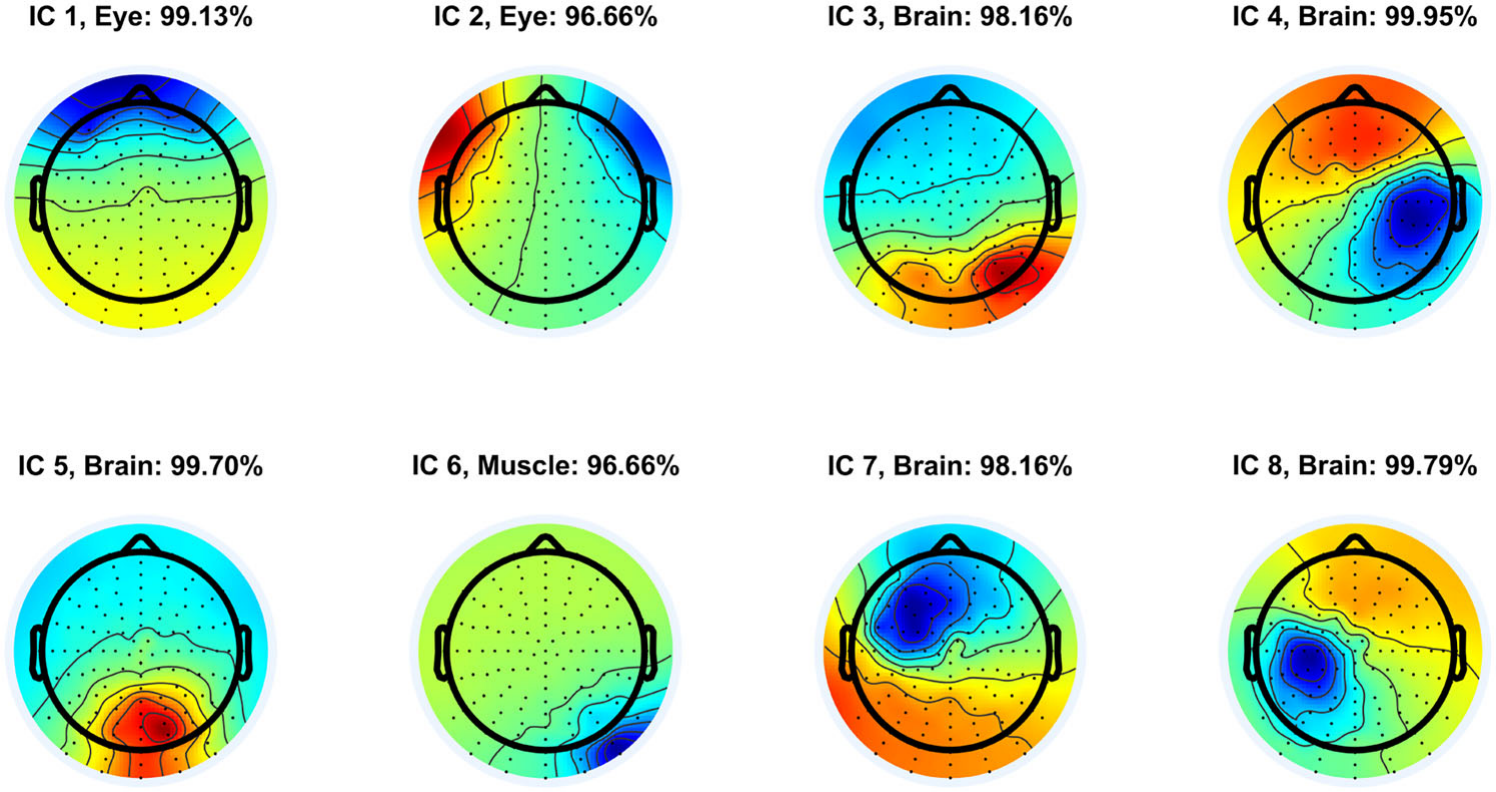
The projected activation maps of the first eight independent components (IC 1–8) of participant S8 with source classes and classification probabilities assigned by the ICLabel algorithm. Components not belonging to the “Brain” class (cortical sources) represent artifacts. The components are sorted in decreasing order by increasing residual variance. See Extended Data Figure 4-1 for electrode layout details.

After executing the pipeline for each participant dataset, two of the authors (experienced investigators) visually inspected all the independent component activations and projected scalp potential maps of the components to check for unique activation patterns and scalp topographies that reflect lambda responses. Figure 4 shows, as an illustration, the first eight independent components of participant S8 sorted by EEGLAB in decreasing order by the mean projected variance of the components. The variance indicates how much a given component accounts for in the EEG data. Components were assigned to source categories by the ICLabel algorithm. Components IC 1 and IC 2 represent eye-related components; IC 1 contains vertical eye movements and blinks, while IC 2 includes horizontal eye movements, respectively. Component IC 5 is a radial dipole located in the occipital region that represents a good candidate for a lambda response component.

10.1523/ENEURO.0270-25.2025.f4-1 Figure 4-1 Biosemi 128-channel ABC layout with O1, Oz, O2 and occipito-parietal electrodes highlighted. Download Figure 4-1, TIF file.

**Figure 5.**
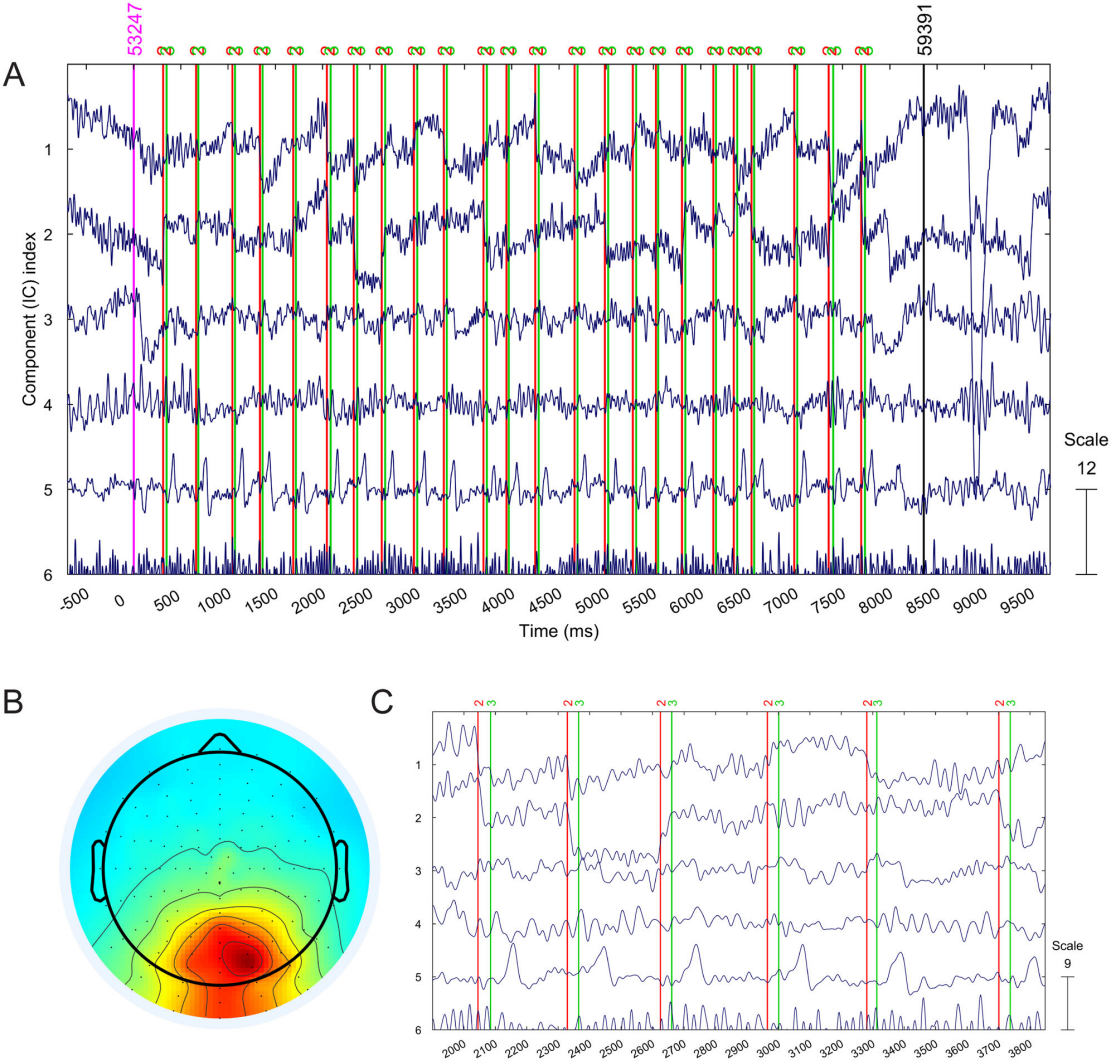
Independent component activations of participant S8 showing the five most important ICs after decomposition. ***A***, One 13 s epoch from the dataset showing distinct triangular waves resembling lambda waveform in independent component 5. IC 1 and 2 are eye components representing blinks and vertical and horizontal movements (their spatial location is shown in Fig. 4). Also displayed are saccade onset (red) and offset (green) events extracted from the eye-tracking data. ***B***, The scalp topography of IC 5 component. ***C***, A zoomed-in section of the component activations showing how the triangular waves regularly follow saccades.

Figure 5*A*, as an illustration, shows the time-dependent activations of the first five Infomax ICA-derived unmixed independent components (ICs) in one epoch of dataset S8. Independent components are sorted by their residual variance $\mathrm{RV}=(\mathrm{var}(\mathrm{EEG})-\mathrm{var}({\mathrm{EEG}}_{k}^{*}))/\mathrm{var}(\mathrm{EEG})$, where var(EEG) is the variance of the original EEG dataset and $\mathrm{var}({\mathrm{EEG}}_{k}^{*})$ is the variance of the EEG dataset back-projected after component $IC_{k}$ was removed. Stimulus (painting display onset) events are marked by red lines while response events (like/dislike button presses) are marked by magenta lines. As in Figure 4, IC 1 and 2 represent blinks and vertical and horizontal eye movements. Note that the fifth component activation (the occipital IC 5) contains a distinct triangular pulse sequence that is present only within the 8 s painting viewing intervals of the 80 paintings but not in the blank-screen response periods. The shape and pattern of the waveform of this component resembles the occipital lambda waves presented in Figures 3 and 4 in Evans (1953). The spatial topography of this component implies that it originates from a central occipital (Oz-POz) region, from a single radial equivalent dipole source that matches the origin of the lambda response (Billings, 1989). Figure 5*C* shows a zoomed-in segment of independent components IC 1–5. The plots also include saccade onset/offset events that were extracted from the eye-tracking measurement and time-synchronized with the EEG data. The spatial activation topography and the saccade-related temporal location of these distinct peaks suggest that this independent component waveform indeed represents the time series of saccade-related lambda responses. The remaining 79 painting epochs show similar triangular spike activation patterns on IC 5. It is important to mention that none of the other 122 independent components showed similar activity and spatial location.

**Figure 6.**
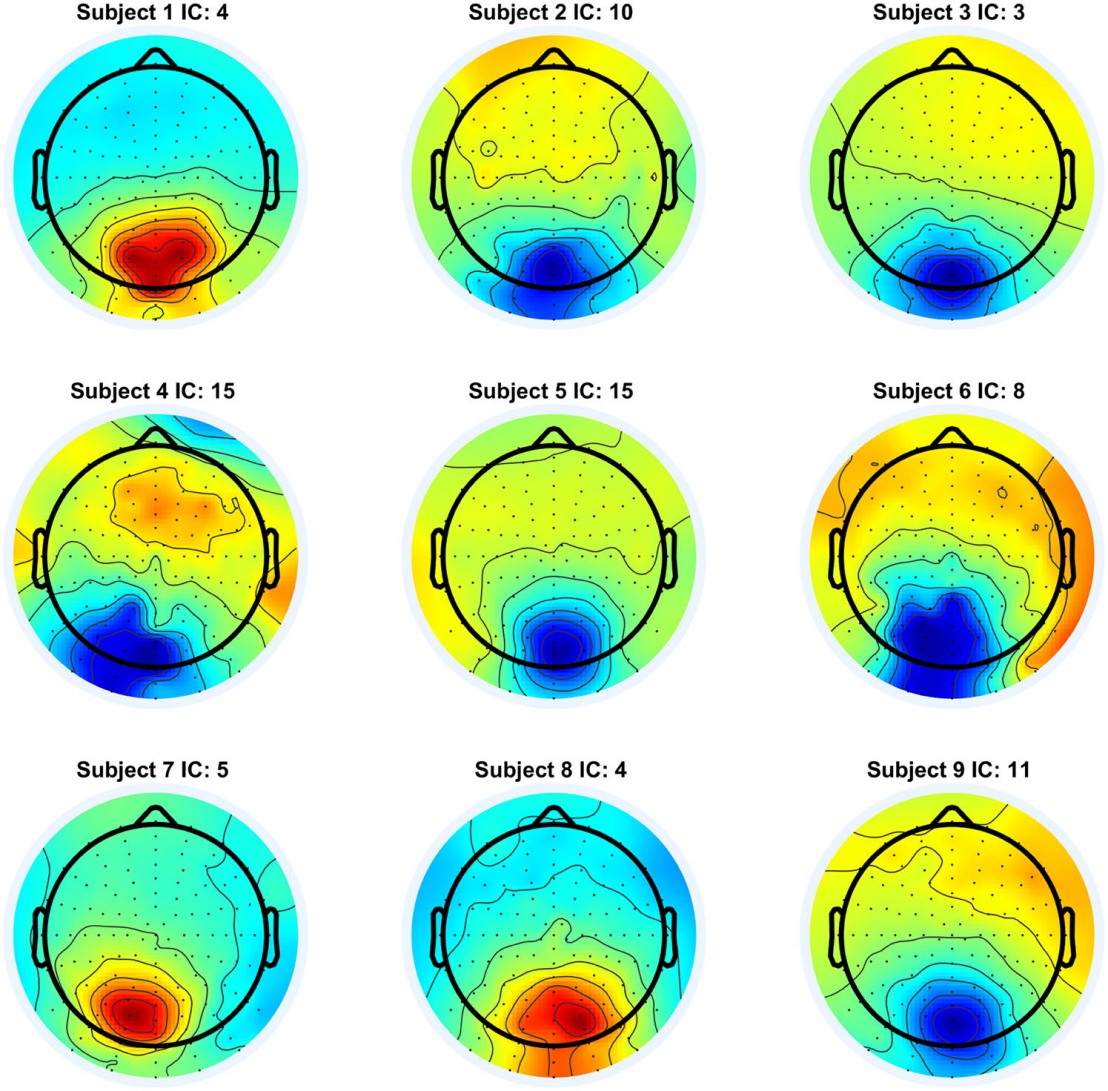
Projected scalp activation maps of the lambda components from each participant.

In Figure 6, we show the participant scalp maps of the identified lambda candidate components. For some subjects, the activation map has negative polarity, which is a result of the sign ambiguity property of ICA (Hyvärinen and Oja, 2000). This does not affect the component in any other ways, only its polarity must be reversed.

The preliminary results suggest that ICA may successfully separate lambda response source activity from other neural and artifact sources. However, to confirm that the extracted waveforms are true lambda responses and that the waveforms are not distorted, we need to perform further validations.

### Lambda component waveform validation

The exploratory analysis steps outlined above, Eye movement data and Exploratory lambda component detection, suggest that the lambda response can be detected with the ICA method as a single independent component. To verify that the extracted component is genuinely the bioelectric source of the lambda response potential measured on the scalp, several additional validation steps are required. First, the triangular waveforms extracted from the candidate lambda components are compared with the averaged occipital potentials locked to saccade onsets and offsets and then to averaged potentials locked to the peak locations of the extracted triangular waveforms (morphology test). Then the saccade onset- and offset-related latencies of the triangular peaks are calculated and matched with results reported in earlier works (timing test). Finally, we create a synthetic lambda response signal and verify the effect of filtering parameters on the detection quality (detection reliability test). The details of each validation steps are outlined below.

#### Component validation by comparison to saccade-related potentials

**Figure 7.**
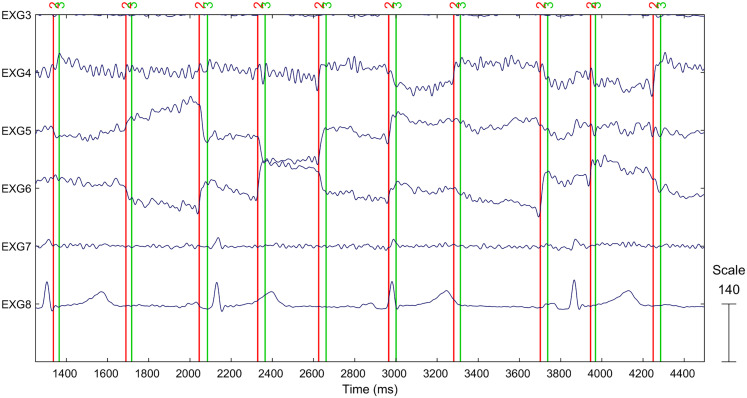
Eye-tracker saccade event (red, onset; green, offset) time aligned to EEG after synchronizing with EOG electrode recordings (EXG5–6 and EXG 4,7—signals from left, right HEOG and top, bottom VEOG electrodes). EXG8 is ECG.

To study the waveforms of the extracted (hypothesized) lambda components, we first epoched the EEG data into 13 s saccade-related epochs including cue, painting, and response sections. Next, the saccade onset/offset latencies that we extracted from the 8 s painting presentation intervals from the eye-tracking registration were imported into the EEG dataset as events. Even though the saccade latencies were exported in 8-s-long epoch-relative time, locked to the start of the painting presentation (*t* = 0 ms), first they had to be converted to continuous time representation used internally by the EEGLAB toolbox. For this, we aligned the *t* = 0 timestamp from the eye-tracking data with beginning of the 8 s painting section in the EEG and then added subsequent eye-tracking data epochs by shifting their event times by a 13 s offset. After this time alignment, the saccade onset and offset timestamps were converted to EEGLAB events and imported into the EEG datasets. As the final step of the eye movement and EEG data time synchronization, we compensated eye-tracker measurement latencies by adjusting saccade events until they were aligned with horizontal and vertical saccades registered by HEOG and VEOG electrodes. The final alignment after a 16 ms shift in saccade events is show in Figure 7.

Following the final saccade event–EEG synchronization step, two EEG datasets were created by re-epoching the 8 s (painting view) sections of the datasets to (−100 to 250 ms) epochs locked to saccade onsets and offsets, respectively. Then, the average potential was calculated from each dataset using all epochs, first for electrodes O1-Oz-O2 (Thickbroom and Mastaglia, 1986; Ries et al., 2018), and then for the occipital electrode that showed the largest amplitude at the time of the lambda response peak for the given participant. The equivalent electrodes of O1, Oz, and O2 in the Biosemi layout are A15, A23, and A28, respectively. Next, the average waveform of the component that was identified as a potential lambda component was calculated for comparison. Details of the lambda component identification are described below, Detection reliability test using real EEG data. To quantify the similarity of the averaged fixation-related potential and the lambda responses, we used the following correlation-based Similarity Index, SI, given as follows:

$$\rho_{i}(x_{i}(t),y_{i}(t))=\frac{\mathrm{cov}(x_{i}(t),y_{i}(t))}{\sqrt{\mathrm{var}(x_{i}(t))}\sqrt{\mathrm{var}(y_{i}(t))}},$$

where cov() is the covariance of the input signals $x_{i}(t)$ and $y_{i}(t)$ and var() is the variance of the individual signals. The index varies between 0 and 1, where ρ=1 means the two signals are identical.

The saccade offset-related potential is identical to the FRP. Since lambda response is assumed to be locked to saccade offsets (i.e., fixation onsets), we expect to obtain higher similarity for saccade offset than onset-related potentials.

#### Lambda peak-locked epoching

**Figure 8.**
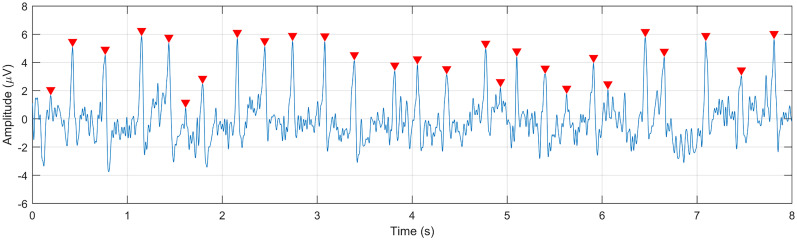
One 8 s epoch of the lambda candidate independent component. Detected peaks that were used as epoching events are marked by red triangles.

Up to this point, following the traditional lambda response computation method, epochs were locked to either the onset or offset of the saccades before averaging the potentials. Even with offset-locked epochs, temporal jitter can result in FRP wave distortions that may have an effect on averaged potential and lambda component waveform comparisons. As the final step of the waveform validation, we re-epoched the 8 s painting view segments into −150 to 150 ms epochs locked to the peak locations of the extracted lambda candidate waves, identified by executing the MATLAB peak detection function

$$[\mathrm{peaks},\mathrm{locs}]=\mathrm{findpeaks}(\mathrm{lambda}\_\mathrm{candidate}\_\mathrm{component},\mathrm{fs}{,}^{\prime }{\mathrm{MinPeakDistance}}^{\prime },\;\;0.14{,}^{\prime }{\mathrm{MinPeakProminence}}^{\prime },3),$$

where lambda_candidate_component is the independent component to be tested, fs = 512 Hz, the minimum peak distance is 150 ms, and the minimum peak prominence is 3 µV. A representative epoch with the detected peaks is shown in Figure 8. The extracted 300-ms-long lambda peak epochs were averaged to compute the average lambda component waves and two average lambda peak-locked occipital potentials (one from the O1-Oz-O2 electrodes and one from the occipito-centroparietal electrode showing the largest amplitude at *t* = 0 ms) to be compared. The advantage of locking to the lambda peaks is that the averaging will less likely distort the temporal accuracy of the peak, and if one is interested in downstream occipital and parietal visual processes, using the lambda peak as a synchronization point may lead to more accurate downstream averaged potential waveforms. The similarity of the averaged occipital potentials and the average lambda waveform are quantified by the similarity index (SI).

#### Lambda peak latency analysis

Lambda responses have a distinct temporal behavior. The lambda response is known to be driven by saccade offsets (Yagi, 1979) producing a distinct monophasic triangular waveform with a peak latency of ∼74 ms after the offset (Thickbroom et al., 1991). When measured from the saccade onset, the lambda peak latency increases with saccade length. Thickbroom et al. (1991) further showed that two extra peaks are also present in the occipital response signal, one at 59 ms and one at 100 ms after saccade onset. To validate that the extracted candidate component follows the lambda temporal pattern, we computed the saccade onset- and offset-locked peak latencies for each epoch of each participant and performed linear regression to test the expected latencies of the component peaks in function of saccade duration.

### Detection reliability test with synthetic ground truth

Besides the question whether ICA could extract a lambda component, a further issue is whether the ICA method can detect lambda waves reliably and without distortion, and whether (and how) the quality of the ICA decomposition depends on preprocessing parameters. The correct characterization of the decomposition quality depends on a known, true cortical lambda source signal (the ground truth) that—unfortunately—cannot be obtained with noninvasive methods. An approximate solution can be achieved using synthetic signals.

**Figure 9.**
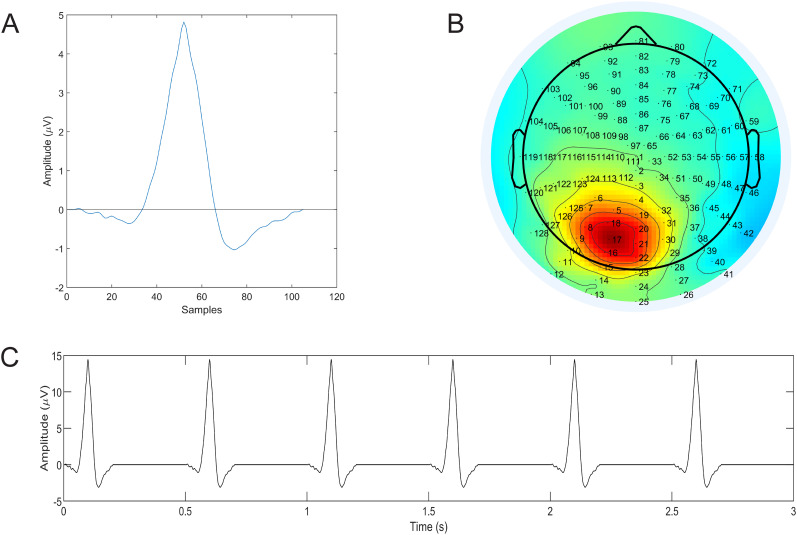
Synthetic lambda ground generation. ***A***, The EEG lambda waveform template, ***B***, map of relative electrode weights for template signal amplitudes, and ***C***, the generated lambda ground truth signal.

We created a synthetic lambda source in order to examine the reliability of its ICA-based extraction as follows. We selected the independent component from the dataset of participant S7 that we assumed to represent a lambda component and epoched it into −50 to 200 ms lambda peak-locked epochs. The epochs were then averaged and from the average waveform we extracted a −50 to 50 ms peak-locked section as a lambda template (shown in Fig. 9*A*) with peak amplitude of 4.82 µV. Using this template after a threefold increase in amplitude, a synthetic lambda sequence was then generated containing 600 copies of the template with 500 ms interpeak distance (Fig. 9*C*). Subsequently, this signal was added to a 5 min open-eyes resting-state EEG dataset. In the resting-state task which was part of the experiment session, participants were asked to look at and fixate on a white wall in front of them, which stimulus is known for not generating lambda responses (Evans, 1953). To simulate the effect of volume conduction, the synthetic lambda signal was added to the resting-state dataset channels with amplitudes modified by the mixing weights $W^{-1}$ obtained from the EEG.icawinv variable in the EEGLAB EEG data structure for the lambda template component. This matrix describes the contribution of the given component to individual EEG channels. The weight pattern used for the synthetic lambda source generation is shown in Figure 9*B*. The maximum peak amplitude of the lambda template signal after applying the weights was 55.42 µV, resulting in a maximum signal-to-noise ratio of SNR = 9.76.

The new, altered resting-state EEG dataset was then preprocessed with different filter parameters to study the effects of filtering on the ICA decomposition result. For this test we used only one dataset at a sampling rate of fs=512Hz. Filter cutoff frequencies HP={0.1,0.5,1,2}HzandLP={40,80,128}Hz were varied separately for the high-pass and low-pass filters, resulting in 12 unique filter parameter combinations. The Infomax ICA algorithm was executed for each of the 12 filtered dataset followed by the computation of the similarity index first (1) between the extracted full-length lambda component and the full-length reference lambda signal (Fig. 9*C*) and then (2) between the lambda template (Fig. 9*A*) and the average lambda peak-locked component waveforms. The first test examines how much noise is left after the ICA decomposition in the component, while the second focuses on how accurately the template waveform was recovered by the ICA step.

### Detection reliability test using real EEG data

Since in real EEG measurements the ground truth signals are not known, the final step in the validation is to test the reliability of the ICA-based detection method on real EEG data using different preprocessing parameter settings. Without a ground truth, we can only compare the ICA-extracted lambda components with one another and study, in an implicit way, how preprocessing parameters affect the quality of the lambda IC extraction. In other words, we check whether ICA can detect the lambda waves in real EEG signals under different parameters and how the parameters affect the shape of lambda waveform. We performed a series of tests in which the following parameters were varied individually; measurement length, EEG sampling rate, and high-pass and low-pass filter cutoff frequencies. Since the length of the dataset is known to affect ICA decomposition quality (Onton et al., 2006), the length of the input dataset L was varied by keeping the first 25, 50, 75, and 100% of the EEG signal samples, represented by parameter L={25,50,75,100}. Sampling rate $f_{s}$ (Hz) was varied by downsampling signals to 1,024 and 512 Hz from the original 2,048 Hz, $f_{s}=\{512,\;1024,\;2048\}$. Filter cutoff frequencies HP, LP (Hz) were varied separately for the high-pass and low-pass filters, HP={0.1,0.5,1,2} and LP={40,80,128}.

We executed Infomax ICA algorithm for the resulting $L\times f_{s}\times \mathrm{HP}\times \mathrm{LP}=144$ preprocessing parameter combinations on the dataset of one participant. Two of the authors with over 15 years of combined EEG processing and analysis experience manually inspected each scalp component map and the ICA activation signals of the resulting datasets and extracted the lambda component candidates when present. Hierarchical cluster analysis (HCA; Murtagh and Contreras, 2012) was used for examining the sensitivity of the lambda component to different parameters. The agglomerative HCA algorithm works by repeatedly combining clusters that show high similarity based on the selected linkage method. Linkage describes the similarity between clusters. First the cross-correlation matrix was computed using pairwise Pearson's correlations. From the correlations the distance metric was derived as 1−|ρ(X,Y)|, such that correlating components have a distance close to zero, while pairs that show no correlation have a distance close to one. The Ward's variance minimization algorithm (Ward, 1963) was used as the linkage method for HCA. Using the SciPy Python package, a dendrogram representation of the HCA was computed and the cluster labels were assigned using the distance threshold selected based on the dendrogram.

### Automatic single-trial lambda detection

Finding the lambda component and the lambda peaks manually after executing ICA on a high-density EEG recording is a tedious, time-consuming, and subjective task. Since the order and the polarity of the extracted independent components can vary from run to run, it is impossible to know in advance which one will contain the expected lambda responses. Here we propose a method that can be used to automatically find components that might represent a lambda response component and on which we should perform lambda waveform detection. The method identifies the lambda component based on its scalp-projected activity pattern. The occipito-parietal area where the lambda activity is expected should be defined a priori as a set of electrodes. In our 128-channel Biosemi layout, these are electrodes within the A15–31 range (Extended Data Fig. 4-1). This step is based on the assumption that expected occipital radial equivalent dipoles should generate a focal activity pattern with a maximum within the occipital region. The key steps of the method are as follows:

Step 1. Perform preprocessing
Step 2. Execute ICA
Step 3. Lambda component identification
  **for** each component IC in the dataset **do**
    - Compute the mean ICA weights of the electrodes in the occipito-parietal area for the current independent component using $W^{-1}$.
    - Find the maximum weight and its corresponding component index
  **endfor**
Step 4. Peak detection
  - Determine the polarity of the component signal based on the mean weight computed in Step 3. Positive weight implies positive lambda peaks in the component, negative weight implies negative lambda peaks.
  - Run peak detection on the component and save the peak locations and amplitudes.

The peak detection in Step 4 can be executed in two different ways depending on whether eye-tracker provided saccade events are available for the analysis. The first method uses saccade information to select only lambda peaks that follow saccade offsets within a 40–140 ms interval. The second method selects lambda peaks without saccade information based on pre-set peak prominence and interpeak distance threshold parameters. The latter uses a higher prominence value to reduce the number of false positive peaks.

#### Lambda peak detection process

1. Extract saccade information from eye movement data (count, onset, and offset time points).
2. Find lambda responses in the component candidate.
  - METHOD A: using eye-tracker data and saccade offset-locked −50 to 200 ms epoched EEG
    - Set peak prominence threshold to 2.
    - Peak detection: find one peak in each epoch
    - If peak found and latency is within 40–140 ms post-offset interval, store the peak location and amplitude information.
  - METHOD B: using continuous or stimulus-locked epoched EEG without eye-tracker data
    - Set peak prominence threshold to 3 and interpeak distance to 140 ms (up to 7 saccades per second).
    - Peak detection: find peaks with the set prominence value.
    - Save the peak location and amplitude information.
3. Saccade—lambda peak matching: select/retain only those detected lambda peaks whose saccade offset-related latency falls within a specified time interval, typically 40–100 ms.
4. Generate EEG events from lambda peak locations and import them together with saccade onsets/offsets into the EEG dataset.

The performance of the detection method is evaluated by the Type I and Type II error in identifying lambda components. Lambda peak extraction performance will be evaluated based on the number of eye-tracker detected valid saccade events and the corresponding number of expected lambda peaks and the number of extracted peaks.

## Results

The exploratory ICA decompositions outlined in Materials and Methods, Exploratory lambda component detection demonstrated that ICA can identify one independent component containing waveforms resembling the lambda wave for each participant. Here we show results of the subsequent validation steps and of the sensitivity analysis of ICA lambda decomposition to different preprocessing parameters.

### Lambda waveform validation

We started the lambda component validation with comparing averaged lambda component waves to saccade onset and offset-related average potentials. Using saccade events exported from the eye-tracker software Tobii Pro Lab and synchronized with the EEG data, we obtained an average 1,797.6 (SD = 261.5) saccades per subject using which we extracted saccade onset- and offset-locked epochs. Epochs containing successive overlapping saccades were excluded. We used subject S7 to compute the similarity measures.

#### Saccade onset-aligned epochs

**Figure 10.**
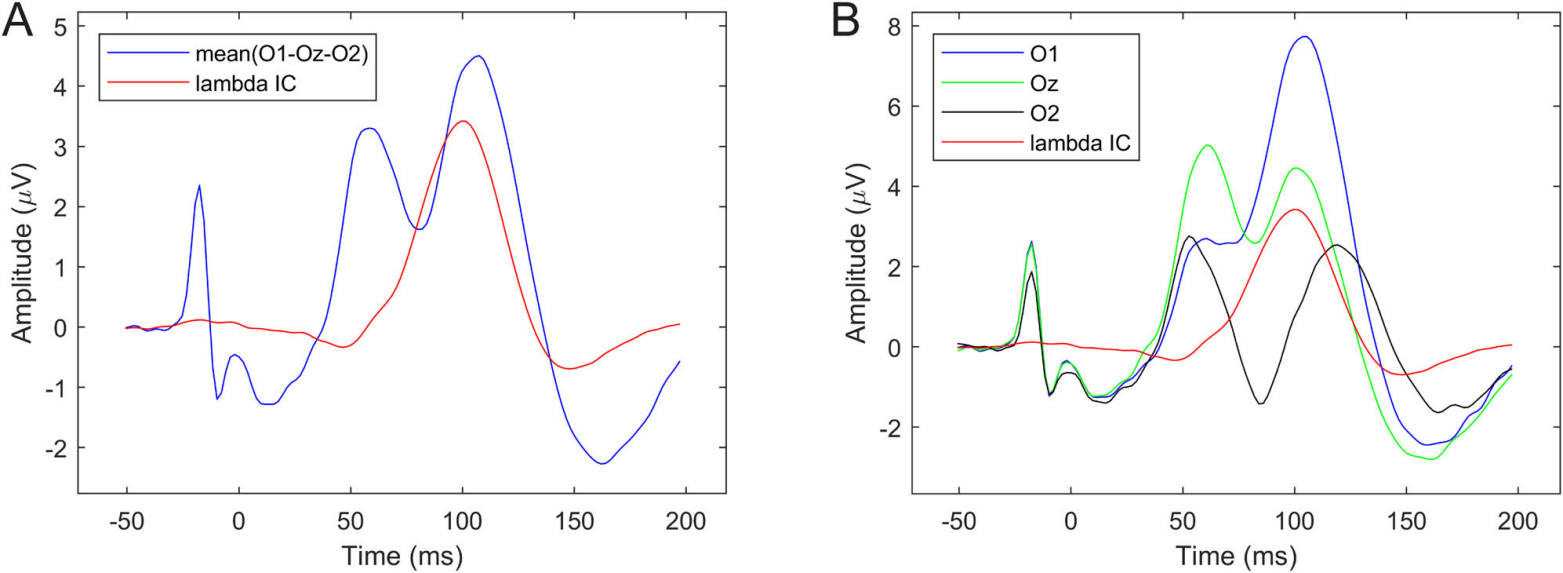
Averaged saccade onset-related potentials versus average lambda component waveforms. ***A***, Averaged O1-Oz-O2 waveform versus lambda waveforms. ***B***, Individual O1, Oz, O2, and lambda waveforms.

First we computed the average potential of O1 (A15), Oz (A23), and O2 (A28) electrodes from the saccade onset-locked epochs and compared it with the averaged lambda IC. Figure 10*A* shows the two average potential waveforms. The average occipital potential clearly shows the saccadic spike potential (SP) preceding the saccade by ∼20 ms, and the two peaks associated with saccade onset- and offset-locked activities. The average potential and lambda waveforms, however, show large differences (different number of peaks and latencies); therefore, in Figure 10*B* we also show the waveforms of the individual occipital electrodes. The similarity index of the three-electrode occipital average and the lambda waveform (based on −50 to 200 ms onset-aligned epochs) was SI = 0.7563.

**Figure 11.**
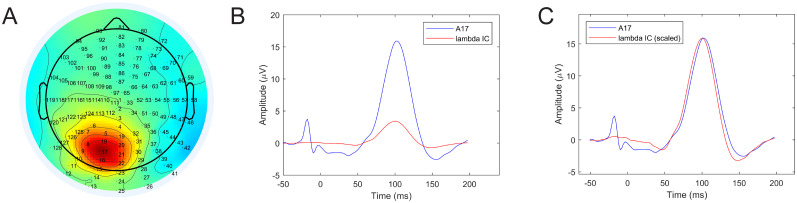
ERP of maximum occipital activity versus average lambda component waveform. ***A***, Scalp activity map showing A17 as peak activation electrode. ***B***, Averaged saccade onset-related A17 waveform versus lambda. ***C***, Same waveforms as in ***B*** but with lambda scaled to match A17 waveform amplitude. See Extended Data Figure 11-1 for saccade onset-locked ERP images of these electrodes.

Next, we selected the electrode from the occipito-parietal region showing the largest activation on the lambda component scalp map (Fig. 11*A*), in this case A17. The maximum occipital electrode ERP and the lambda waveforms are shown in Figure 11*B*. The plot in Figure 11*C* shows the lambda component scaled to the A17 potential peak for better visual similarity assessment. The increased similarity of the waveform shapes and the better alignment of the dominant peaks of the two signals compared with the O1-Oz-O2 electrodes is clearly visible. The similarity index of the A17 electrode average and the lambda wave is SI = 0.9759.

10.1523/ENEURO.0270-25.2025.f11-1 Figure 11-1 Saccade onset locked ERP images of channel O1 (A15), Oz (A23), O2 (A28), average of O1-Oz-O2 and A17 vs. IC5 (lambda independent component) epochs sorted by saccade duration measured on participant S7. Other participants show similar results. The occipital ERPs show the saccadic spike potential SP (-20 ms) and two distinct peaks with varying amplitudes. The first peak follows saccade onset by ∼ 50 ms, the second peak follows saccade offset by ∼100 ms. The average ERP waveform (blue plot at the bottom of each pane) cannot depict the temporal jitter hence the behavior of the offset-locked peak latencies. Download Figure 11-1, TIF file.

#### Saccade offset-aligned epochs

**Figure 12.**
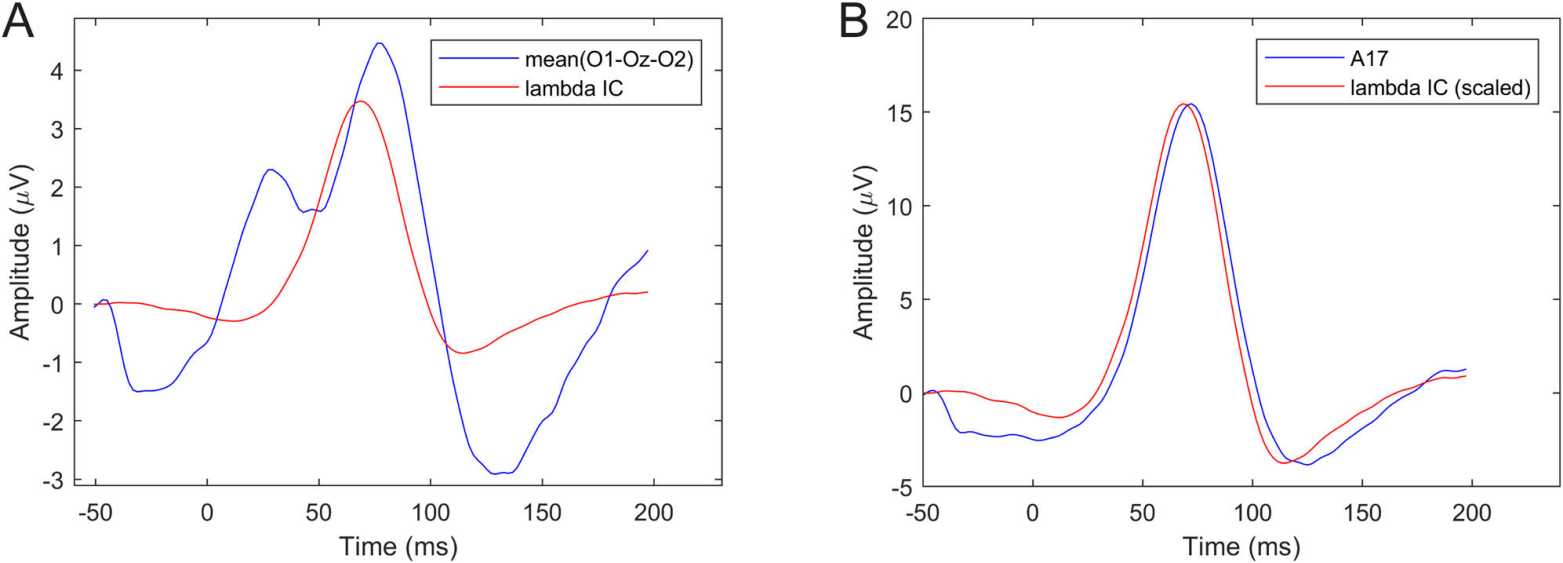
Averaged saccade offset-related potentials versus average lambda component waveforms. ***A***, Averaged O1-Oz-O2 waveform versus lambda. ***B***, Averaged saccade offset-related A17 waveform versus scaled lambda signal. See Extended Data Figure 12-1 for saccade offset-locked ERP images of these electrodes.

The second stage of the waveform validation was based on saccade offset epochs, since lambda response is assumed to be generated by saccade offsets (fixation onsets; Yagi, 1979). Following the same epoching and averaging procedure, we extracted the average O1-Oz-O2 FRP, the A17 FRP locked to saccade offsets and compared them with the offset-locked candidate average lambda wave independent component signal. Both cases are illustrated in Figure 12. The similarity index is SI = 0.7934 for the average occipital potentials and SI = 0.9771 for the A17 electrode FRP. Just as with the saccade onset-locked case, the averaged occipital FRP shows waveform and peak latency differences. The A17 electrode FRP, however, is more accurately aligned with the average of the assumed lambda component confirming that the waveforms in the independent component are indeed lambda waveforms.

10.1523/ENEURO.0270-25.2025.f12-1 Figure 12-1 Saccade offset locked ERP images of channel O1 (A15), Oz (A23), O2 (A28), average of O1-Oz-O2 and A17 vs. IC5 (lambda independent component) epochs sorted by saccade duration measured on participant S7. The second (offset locked) peak shows saccade onset influence; the onset of longer saccades moves the location of the offset-locked second peak slightly forward in time (most prominent at O1 and Oz). Download Figure 12-1, TIF file.

#### Lambda peak-locked epochs

**Figure 13.**
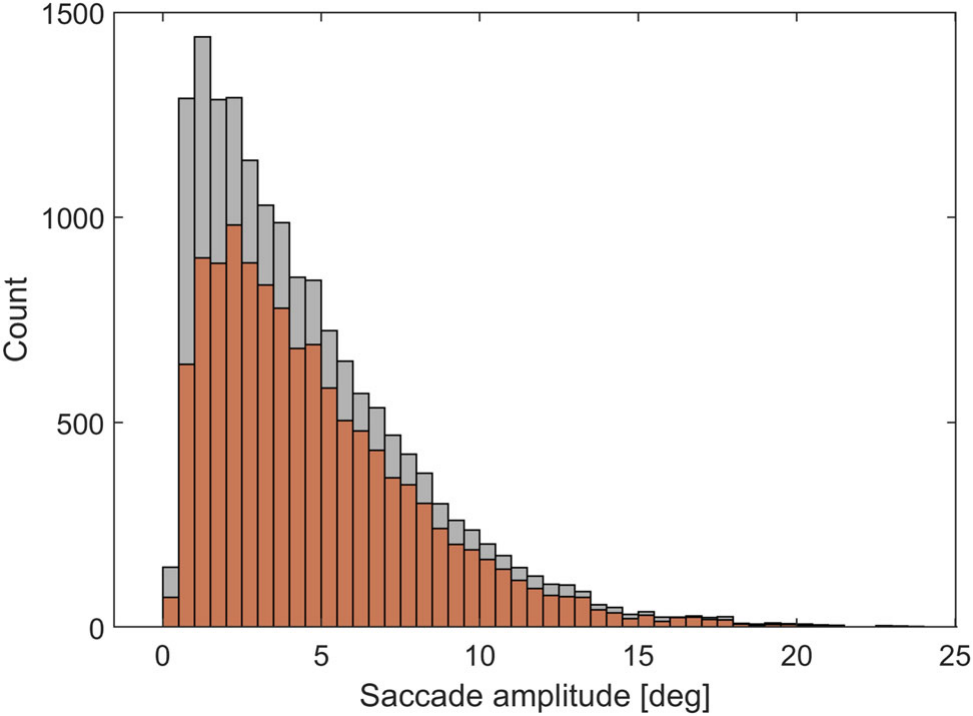
Distribution of saccades (gray) and detected matching lambda peaks (orange).

The final stage of the lambda waveform analysis was to compare occipital average potentials with the lambda component based on epochs locked (−50 to 250 ms) not to saccades onsets or offsets, but to lambda peaks. For participant S7, we found 1,593 saccades and detected 1,906 lambda peaks. After matching saccades with following lambda peaks, we obtained 1,252 (78.6%) saccade–peak pairs. These lambda peaks could be used for epoching the EEG signal locked to the lambda peak. The distribution of the saccades and the verified lambda peaks are shown in Figure 13. The distribution confirms that the detection of individual lambda waveforms is independent of saccade amplitude.

**Figure 14.**
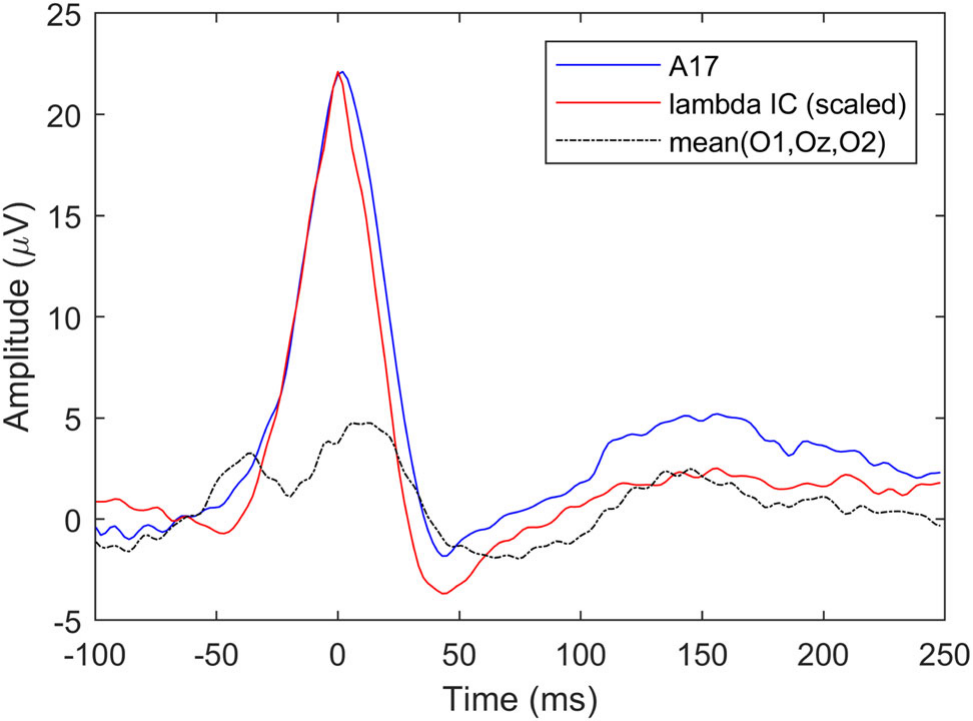
The average occipital O1-Oz-O2 and A17 lambda peak-locked ERPs versus the average ICA-detected lambda wave.

The averaged potential and lambda component ERP results, shown in Figure 14, are similar to the previous two cases. Electrode A17 average potential shows the highest similarity with the average lambda. The best alignment between A17 and lambda is in the −50 to 100 ms interval. The similarity index is SI = 0.6857 for the average occipital potentials and SI = 0.9693 for the A17 electrode FRP. Note that the peak at *t* = 0 is much sharper than the lambda peaks in the saccade onset- and offset-locked cases (on Figs. 11, 12) due to no temporal jitter effect on the peak during averaging.

The similarity index values for the three cases and two different electrode waveforms are listed in Table 1. We also computed the similarity index limited to −50 to 50 ms interval around the lambda peak. These values are listed in brackets in the table.

**Table 1.** Waveform similarity results for full saccade-related (−50 to 200 ms) and lambda peak-locked (−100 to 250 ms) epochs

	Saccade onset-locked epochs	Saccade offset-locked epochs	Lambda peak-locked epochs
Avg (O1, Oz, O2) versus lambda IC	0.7563 (0.7137)	0.7934 (0.7854)	0.6954 (0.6922)
A17 versus lambda IC	0.9759 (0.9791)	0.9771 (0.9781)	0.9725 (0.9862)

Similarity values obtained with comparing for the lambda template only (−50 to 50 ms) are in brackets.

The waveform validation results indicate that best match for the lambda component was achieved with the lambda peak-locked average A17 potentials and the offset-related lambda peak latency falls within the 60–80 ms interval following saccade offset, where lambda response is normally expected. The results also confirm that occipital ERPs/FRPs are a mixture of several occipital processes and selecting Oz or O1/O2 electrode could be a suboptimal choice for studying lambda responses as the use of standard fix locations is insensitive to individual variations of cortical anatomy and/or in electrode cap placements.

### Lambda peak latency

**Figure 15.**
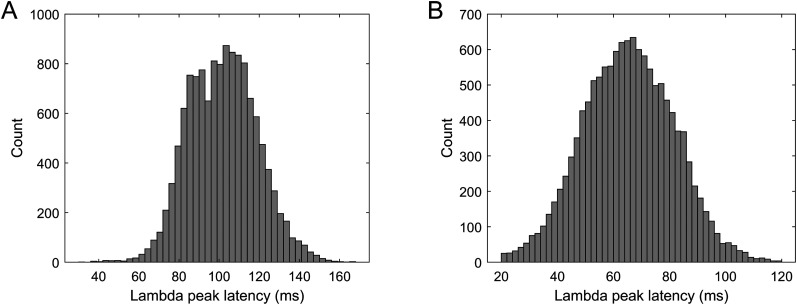
Distribution of the saccade (***A***) onset- and (***B***) offset-related lambda IC peak latency values.

Using the detected lambda component peaks and the matched saccade onset and offset timestamps, we computed the onset- and offset-related lambda IC peak latencies for each epoch and for each participant. The mean onset-related lambda peak latency is 101.3250 (SD = 16.9786), while the offset-related mean latency is 64.8566 (SD = 16.1347). The distributions of the two variables are shown in Figure 15. These results confirm lambda IC peak latency values showed during FRP and lambda waveform comparisons.

**Figure 16.**
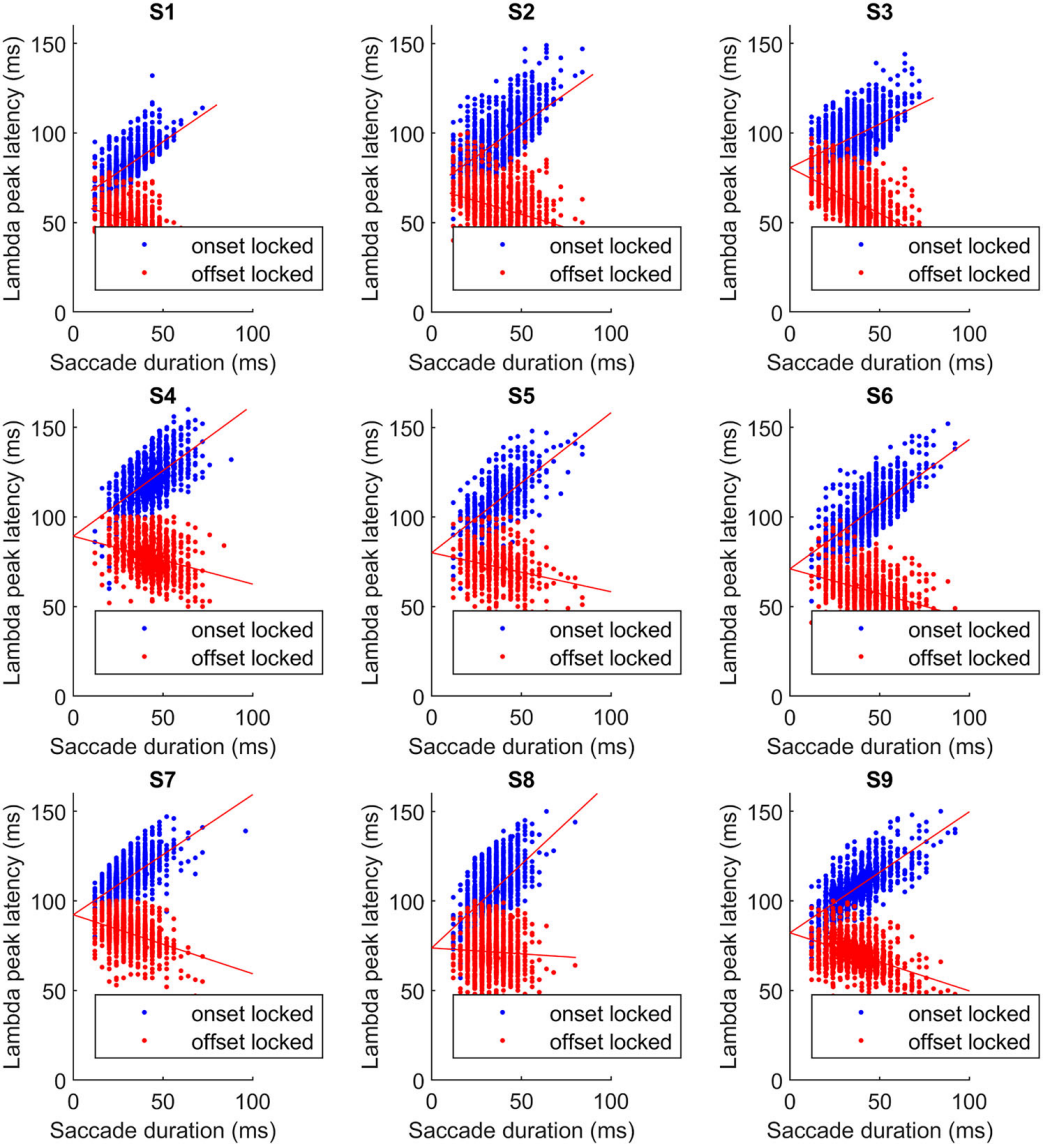
Saccade onset (blue markers) and offset-related (red markers) lambda peak latency values in function of saccade duration with regression lines (red line) fitted to onset and offset latencies indicating their dependencies on saccade duration.

The distribution of saccade onset- and offset-related lambda peak latency values as a function of saccade duration is plotted in Figure 16 for each participant. We also fitted trend lines with linear regression to detect the effect of saccade duration on onset- and offset-related latencies. The detailed results of the regressions are listed in Table 2.

**Table 2.** Saccade onset and offset-locked lambda peak latency linear regression coefficients

Participant	Onset-locked line fit *a*x + *b*		Offset-locked line fit *a*x + *b*	
	*a* (conf. int.)	*b* (conf. int.)	*a* (conf. int.)	*b* (conf. int.)
S1	0.68 (0.65, 0.72)	60.97 (59.81, 62.12)	−0.31 (−0.35, −0.28)	60.97 (59.81, 62.12)
S2	0.70 (0.66, 0.75)	69.38 (67.6, 71.15)	−0.30 (−0.34, −0.25)	69.38 (67.6, 71.15)
S3	0.49 (0.45, 0.52)	80.64 (79.34, 81.93)	−0.51 (−0.55, −0.48)	80.64 (79.34, 81.93)
S4	0.73 (0.68, 0.79)	89.23 (86.91, 91.55)	−0.27 (−0.32, −0.21)	89.23 (86.91, 91.55)
S5	0.78 (0.72, 0.85)	80.01 (77.56, 82.47)	−0.22 (−0.28, −0.15)	80.01 (77.56, 82.47)
S6	0.72 (0.69, 0.76)	71.05 (69.47, 72.62)	−0.28 (−0.31, −0.24)	71.05 (69.47, 72.62)
S7	0.67 (0.623, 0.71)	92.26 (90.85, 93.67)	−0.33 (−0.37, −0.29)	92.26 (90.85, 93.67)
S8	0.93 (0.87, 0.99)	73.73 (71.62, 75.83)	−0.07 (−0.13, −0.01)	73.73 (71.62, 75.83)
S9	0.68 (0.64, 0.71)	82.19 (80.77, 83.61)	−0.32 (−0.36, −0.29)	82.19 (80.77, 83.61)

Confidence intervals of the coefficients are given within brackets.

The results show individual variability in several aspects. The average latency of the lambda peak for most subjects is ∼70–80 ms. For some subjects the average is close to 100 ms, while for one subject it is <60 ms. The general trend is that the onset–offset latency value pairs widen as the saccade duration increases. This is in agreement with previous literature. However, we expected fix latency from offset and increasing latency from onset as shown in Thickbroom et al. (1991). Our data show a slightly different pattern; all subjects, except S8, show an offset latency trend that is decreasing with increasing saccade duration.

### Ground truth lambda detection

**Figure 17.**
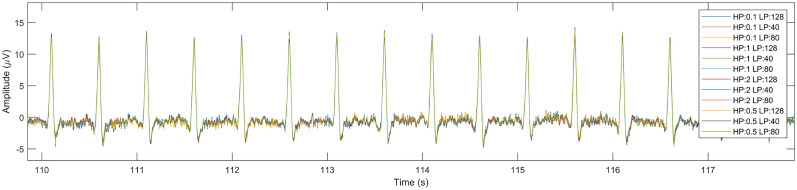
The lambda components extracted from the 12 different resting-state synthetic ground truth datasets.

**Figure 18.**
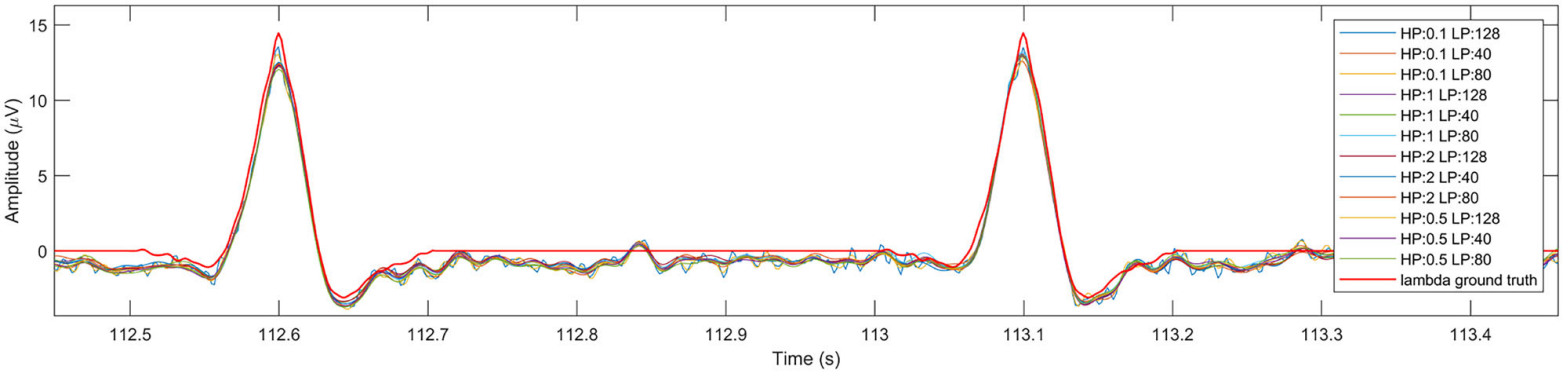
A two-peak segment of the extracted components with the lambda ground truth (red line).

The next step of our investigation aimed at testing whether the extraction of lambda components by ICA depends on preprocessing parameters and how these parameters affect the shape of the extracted lambda waveform. Using the synthetic dataset described in Materials and Methods, Lambda peak latency analysis, we executed the ICA steps for 12 different high-pass and low-pass filter cutoff frequency combinations. The extracted components were compared with the synthetic ground truth lambda signal (Fig. 9*C*). A section of the resulting extracted components from the 12 different parameter runs are plotted in Figure 17. For illustration purposes, we also show a more detailed of the extracted components in Figure 18 with the reference lambda signal.

Similarity between the ground truth lambda signal and the extracted components were calculated in two different ways. The first method compared the full-length lambda signal (600 lambda peaks) and the full-length extracted lambda components. The similarity index values from this comparison are listed in Table 3. This comparison aimed at characterizing the amount of remaining noise and source mixing mainly within the interpeak intervals of the signals.

**Table 3.** Similarity values of full ground truth lambda signal and lambda components extracted under different filtering parameters

LP cutoff (Hz)	HP filter cutoff (Hz)			
	0.1	0.5	1	2
40	0.975	0.970	0.954	0.941
80	0.977	0.968	0.960	0.887
128	0.979	0.967	0.955	0.940

**Figure 19.**
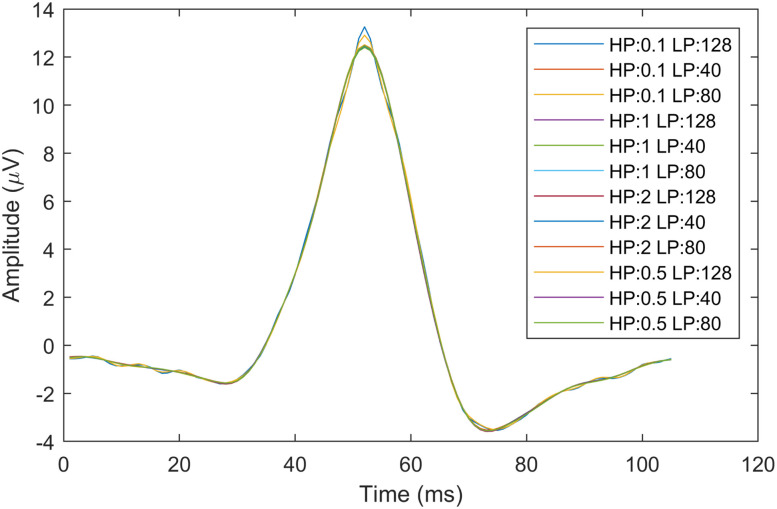
The extracted averaged lambda waveforms obtained with different preprocessing parameters.

The second method compared a single wave template (Fig. 9*A*) as ground truth and the averaged lambda peaks of each parameter dependent extracted lambda independent component shown in Figure 19. The corresponding similarity index values of this test are listed in Table 4.

**Table 4.** Similarity values of the lambda template and the averaged lambda component waveforms extracted under different filtering parameters

LP cutoff (Hz)	HP filter cutoff (Hz)			
	0.1	0.5	1	2
40	0.99932	0.99924	0.99921	0.99919
80	0.99981	0.99930	0.99925	0.99921
128	0.99994	0.99930	0.99923	0.99917

The results indicate that when taking the full signal into consideration, the best result is achieved with HP = 0.1 Hz, with gradually reducing similarity values as we increase the high-pass cutoff frequency up to 2 Hz. The similarity shows minimal effect for low-pass filter parameters. The individual lambda waveform comparisons show similar trend, with the best match obtained with HP = 0.1 Hz and LP = 128 Hz filter parameters, suggesting that waveform distortion can be expected with increasing high-pass and low-pass filtering, as we remove more low- and high-frequency details from the lambda wave. In summary, all high-pass and low-pass filter frequency combinations resulted in ICA-decomposed lambda components.

### Preprocessing effects on lambda detection in real EEG

**Figure 20.**
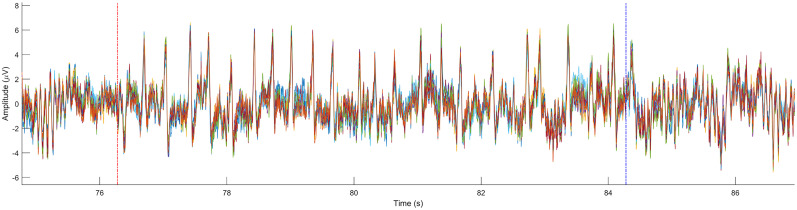
One epoch of the extracted lambda components obtained with different preprocessing settings. Epoch onset is marked by the red vertical line, while the offset is by the blue vertical line.

Following the ground truth analysis, we investigated the preprocessing effects on ICA-based lambda detection using real EEG data. The reliability of the ICA lambda detection was tested executing the Infomax ICA after preprocessing with the following parameter combinations: sampling rate $f_{s}=\{512,\;1024,\;2048\}\mathrm{Hz}$, high-pass and low-pass cutoff frequencies, HP={0.1,0.5,1,2}Hz, LP={40,80,128}Hz, and the signal length L={25,50,75,100}%. The execution of the 4×4×3×4=144 combinations took ∼160 h. The HP=0.1Hz high-pass filtered datasets (*N* = 36) failed to produce a lambda component; hence, they were excluded from the subsequent sensitivity analysis. Figure 20 shows one epoch of the 108 (75%) successfully extracted lambda components.

**Figure 21.**
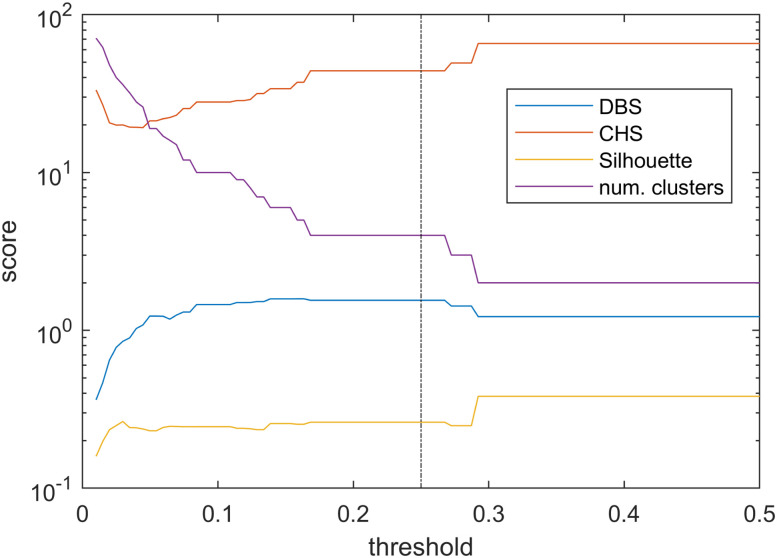
Clustering parameters changing in function of cluster distance metric threshold.

The sensitivity analysis of the extracted lambda components were performed using HCA. The results of the HCA were analyzed by evaluating the Silhouette score (Rousseeuw, 1987), Calinski–Harabasz Score (CHS) (Caliñski and Harabasz, 1974), and Davies–Bouldin Score (DBS; Davies and Bouldin, 1979) model-performance metrics. The scores also allowed the tuning of the selected distance threshold to achieve better separated clusters. This was systematically done with a script iterating over the distance threshold value starting from zero and going up to the point where only two clusters were left. The scores were calculated and plotted for each threshold as shown in Figure 21. Aiming for low DBS, high CHS and close to one Silhouette scores (Silhouette score = 0.2619, CHS = 44.1195, DBS = 1.55), the final threshold of 0.25 was selected resulting in four clusters.

**Figure 22.**
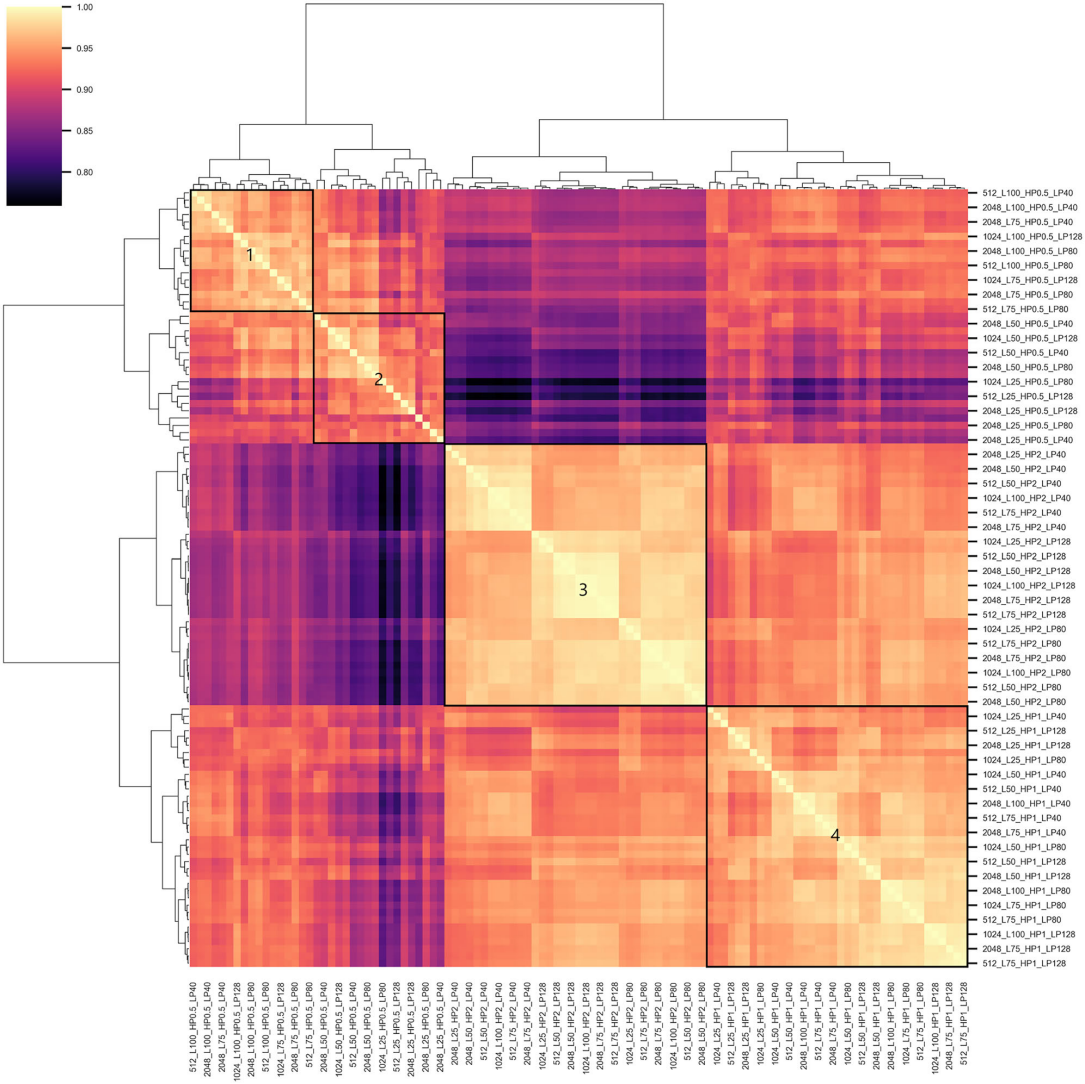
Visual representation of the hierarchical clustering result. Outlined squares numbered 1–4 represent the four clusters. Lighter colors indicate higher similarity between clusters and parameters. See Extended Data Figure 22-1 for the projected scalp maps of the lambda component obtained with the 108 different parameter combinations.

The final four clusters, the similarity values, and the cluster dendrogram are shown in Figure 22. Colors indicate correlation values in the range of 0.759–0.999 (diagonal values are ignored). The mean correlation is 0.918 (SD = 0.049). The mean within-cluster correlation means are *µ*_1_ = 0.958 (SD = 0.014), *µ*_2_ = 0.935 (SD = 0.023), *µ*_3_ = 0.973 (SD = 0.013), and *µ*_4_ = 0.963 (SD = 0.017) for Clusters 1–4, respectively, indicating that Cluster 3 is the most uniform cluster, followed by Cluster 4 and then by Cluster 1.

10.1523/ENEURO.0270-25.2025.f22-1 Figure 22-1 Projected scalp maps of the extracted component after performing automatic lambda identification on the 108 HP {greater than or equal to} 0.5 Hz parameter combination pre-processed datasets. Download Figure 22-1, TIF file.

The parameter combinations belonging to each cluster are listed in Table 5. Cluster 1 contains HP = 0.5 and larger than 50% data length values. Cluster 2 contains the HP = 0.5 Hz parameters with 25 and 50% data length values. Cluster 3 contains all the HP = 2 Hz, while Cluster 4 contains all HP = 1 Hz high-pass filter-based parameter combinations. The result indicates that high-pass filtering plays the most important role in lambda detection performance followed by data length. Sampling frequency and low-pass filtering have lower effect on the outcome.

**Table 5.** Parameter combinations clustered into four groups based on correlation similarity

	Cluster 1	Cluster 2	Cluster 3	Cluster 4
1	1024_L100_HP0.5_LP128	1024_L25_HP0.5_LP128	1024_L100_HP2_LP128	1024_L100_HP1_LP128
2	1024_L100_HP0.5_LP40	1024_L25_HP0.5_LP40	1024_L100_HP2_LP40	1024_L100_HP1_LP40
3	1024_L100_HP0.5_LP80	1024_L25_HP0.5_LP80	1024_L100_HP2_LP80	1024_L100_HP1_LP80
4	1024_L75_HP0.5_LP128	1024_L50_HP0.5_LP128	1024_L25_HP2_LP128	1024_L25_HP1_LP128
5	1024_L75_HP0.5_LP40	1024_L50_HP0.5_LP40	1024_L25_HP2_LP40	1024_L25_HP1_LP40
6	1024_L75_HP0.5_LP80	1024_L50_HP0.5_LP80	1024_L25_HP2_LP80	1024_L25_HP1_LP80
7	2048_L100_HP0.5_LP128	2048_L25_HP0.5_LP128	1024_L50_HP2_LP128	1024_L50_HP1_LP128
8	2048_L100_HP0.5_LP40	2048_L25_HP0.5_LP40	1024_L50_HP2_LP40	1024_L50_HP1_LP40
9	2048_L100_HP0.5_LP80	2048_L25_HP0.5_LP80	1024_L50_HP2_LP80	1024_L50_HP1_LP80
10	2048_L75_HP0.5_LP128	2048_L50_HP0.5_LP128	1024_L75_HP2_LP128	1024_L75_HP1_LP128
11	2048_L75_HP0.5_LP40	2048_L50_HP0.5_LP40	1024_L75_HP2_LP40	1024_L75_HP1_LP40
12	2048_L75_HP0.5_LP80	2048_L50_HP0.5_LP80	1024_L75_HP2_LP80	1024_L75_HP1_LP80
13	512_L100_HP0.5_LP128	512_L25_HP0.5_LP128	2048_L100_HP2_LP128	2048_L100_HP1_LP128
14	512_L100_HP0.5_LP40	512_L25_HP0.5_LP40	2048_L100_HP2_LP40	2048_L100_HP1_LP40
15	512_L100_HP0.5_LP80	512_L25_HP0.5_LP80	2048_L100_HP2_LP80	2048_L100_HP1_LP80
16	512_L75_HP0.5_LP128	512_L50_HP0.5_LP128	2048_L25_HP2_LP128	2048_L25_HP1_LP128
17	512_L75_HP0.5_LP40	512_L50_HP0.5_LP40	2048_L25_HP2_LP40	2048_L25_HP1_LP40
18	512_L75_HP0.5_LP80	512_L50_HP0.5_LP80	2048_L25_HP2_LP80	2048_L25_HP1_LP80
19			2048_L50_HP2_LP128	2048_L50_HP1_LP128
20			2048_L50_HP2_LP40	2048_L50_HP1_LP40
21			2048_L50_HP2_LP80	2048_L50_HP1_LP80
22			2048_L75_HP2_LP128	2048_L75_HP1_LP128
23			2048_L75_HP2_LP40	2048_L75_HP1_LP40
24			2048_L75_HP2_LP80	2048_L75_HP1_LP80
25			512_L100_HP2_LP128	512_L100_HP1_LP128
26			512_L100_HP2_LP40	512_L100_HP1_LP40
27			512_L100_HP2_LP80	512_L100_HP1_LP80
28			512_L25_HP2_LP128	512_L25_HP1_LP128
29			512_L25_HP2_LP40	512_L25_HP1_LP40
30			512_L25_HP2_LP80	512_L25_HP1_LP80
31			512_L50_HP2_LP128	512_L50_HP1_LP128
32			512_L50_HP2_LP40	512_L50_HP1_LP40
33			512_L50_HP2_LP80	512_L50_HP1_LP80
34			512_L75_HP2_LP128	512_L75_HP1_LP128
35			512_L75_HP2_LP40	512_L75_HP1_LP40
36			512_L75_HP2_LP80	512_L75_HP1_LP80

**Figure 23.**
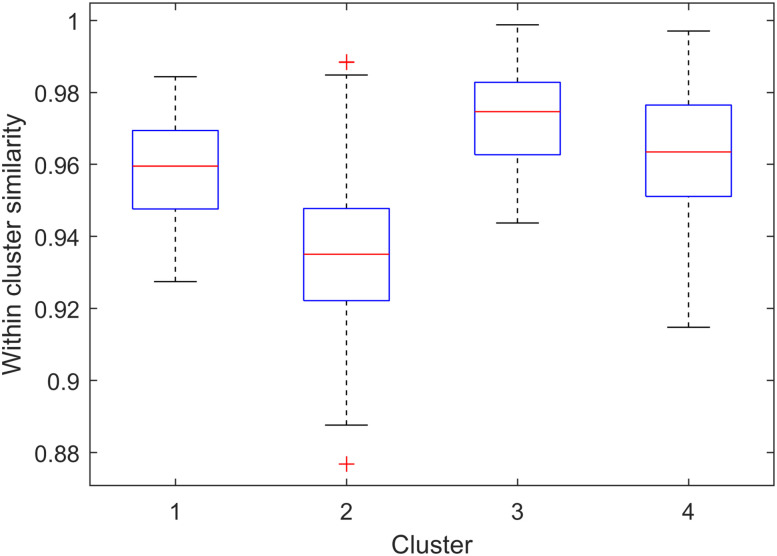
Distribution of within-cluster similarity values. On each box, the red mark indicates the median, and the bottom and top edges of the box indicate the 25th and 75th percentiles, respectively. The whiskers extend to the most extreme data points not considered outliers, and the outliers are plotted individually using the “+” marker symbol.

We performed the Kruskal–Wallis test to test for differences among the four clusters. The results ( $\chi^{2}=374.467,\;p<0.001$; Fig. 23, Table 6) indicate that all groups came from different distributions, confirming the correctness of the clustering step. The *t* test-based multiple-comparison test with Tukey–Kramer correction was applied to test the differences between each cluster. The results, listed in Table 7, indicate that each cluster is different from the others.

**Table 6.** ANOVA table of the Kruskal–Wallis test results

Source	SS	Degrees of freedom	MS	Chi-sq	Prob > χ^2^
Groups	74,923,087	3	24,974,362	374.4672	7.50 × 10^−81^
Error	2.35 × 10^8^	1,545	151,973.8	[]	[]
Total	3.1 × 10^8^	1,548	[]	[]	[]

**Table 7.** Multiple-comparison test results for the clusters

Group A	Group B	Lower limit	A-B	Upper limit	*p* value
1	2	207.5121	342.9387	478.3654	4.48 × 10^−10^
1	3	−494.61	−385.956	−277.302	9.16 × 10^−20^
1	4	−231.976	−123.322	−14.6686	0.018616
2	3	−832.465	−728.894	−625.324	0
2	4	−569.831	−466.261	−362.691	0
3	4	197.887	262.6333	327.3797	0

### Automatic lambda component detection

To help practitioners in detecting lambda components of multiple subjects in a reproducible and objective way, we proposed an automated method in Materials and Methods, Automatic single-trial lambda detection for (1) identifying the independent component that contains lambda peaks and (2) can detect the peaks in the identified lambda component. We have executed the component identification step on the 108 parameter study datasets high-pass filtered at 0.5 Hz and above. The method correctly identified (True Positives, TP = 100%) a lambda component in all cases (Extended Data Fig. 22-1).

### Lambda wave extraction

**Figure 24.**
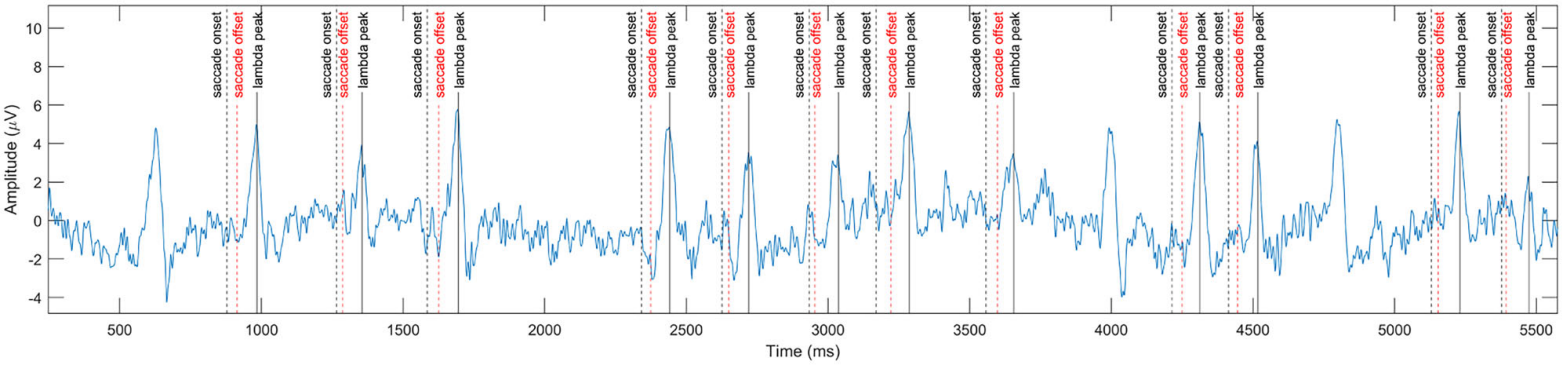
Illustration of the ICA-detected lambda waves (positive-going peaks in blue) with matching saccade onsets and offsets marked by black and red dashed vertical lines, respectively. Three potential lambda peaks could not be matched with saccades as the corresponding saccades were not registered by the eye-tracker.

The second stage of the automatic detection is the extraction of individual lambda peaks from the lambda component by peak finding. We report in Table 8 the results of the eye-tracker combined peak detection method (Method A), in which saccade offsets are matched with subsequent lambda peaks, and in Table 9 (Method B), where the peak detection is performed without saccade information based on prominence and interpeak distance thresholds. The achieved mean peak detection rate of Method A was 85.42% (SD = 9.286). There were missing saccade–lambda pairs, due to either saccades undetected by the eye-tracker or very small amplitude or late lambda peaks after the saccade (see Fig. 24 for illustration). The mean peak prominence of the peaks detected with Method A was 4.58, based on which we set the minimum peak prominence to 3 in Method B, and used a minimum interpeak distance of 140 ms. The detection was performed on the 8 s painting view segments only.

**Table 8.** Lambda peak extraction results of the saccade-assisted peak detection (Method A)

Participant	Num. eye-tracker saccades	Num. saccade offset epochs	Num. detected saccade–lambda pairs (%)
S1	2,210	2,157	1,753 (81.3%)
S2	1,903	1,868	1,481 (79.3%)
S3	2,052	2,013	1,919 (95.3%)
S4	2,034	1,984	1,592 (80.2%)
S5	1,462	1,417	950 (67.0%)
S6	1,660	1,625	1,429 (87.9%)
S7	1,593	1,560	1,200 (76.9%)
S8	1,724	1,688	1,611 (95.4%)
S9	1,540	1,503	1,375 (91.5%)

**Table 9.** Lambda peak extraction results obtained without saccade information (Method B)

Participant	Num. eye-tracker saccades	Num. detected lambda peaks
S1	2,210	2,718
S2	1,903	2,158
S3	2,052	2,411
S4	2,034	1,469
S5	1,462	1,167
S6	1,660	1,730
S7	1,593	1,906
S8	1,724	2,021
S9	1,540	1,921

### Saccade-related occipital processes

**Figure 25.**
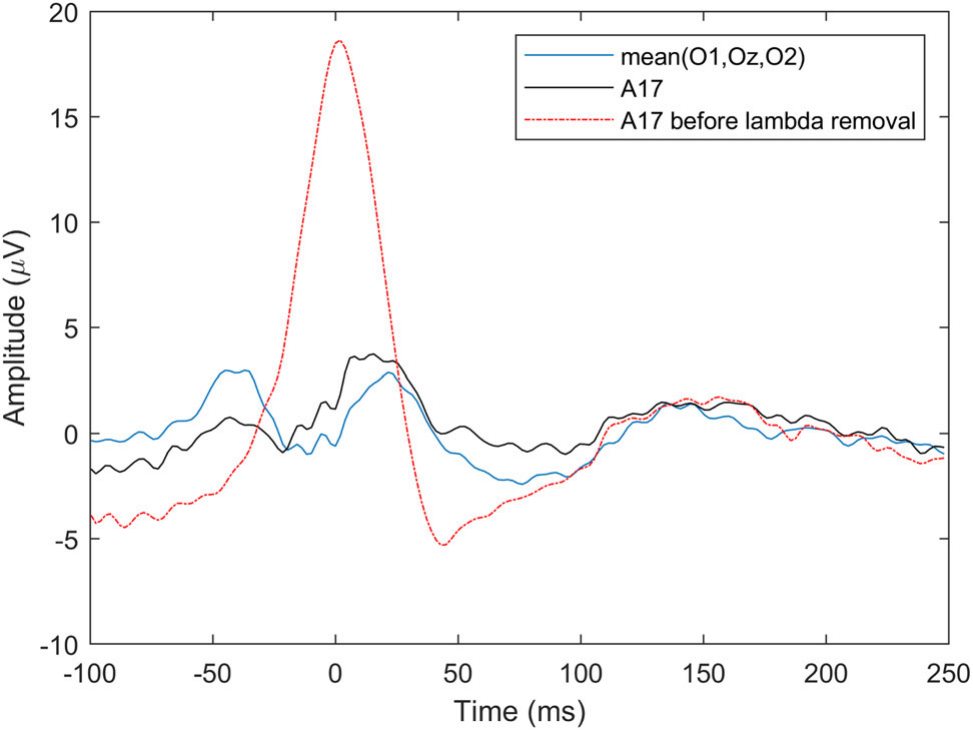
The lambda peak-locked ERP potentials of two different areas (O1-Oz-O2 vs A17) obtained after removing the lambda independent component from the EEG data. The remaining activities of these two signal groups show more similarities than before lambda removal.

In free-viewing experiments, the fixation-related lambda response dominates average potentials. The extracted lambda component, as an independent component, facilitates the removal of lambda activity from the averaged potential in situations where saccade-related activities are considered as artifacts. The lambda waveform validation results showed that for participant S7, channel A17 was most similar to the lambda response, not the O1, Oz, and O2 channels. In Figure 25, we show the effect of removing the lambda component on two average occipital potentials (O1-Oz-O2 and A17), but this time with the lambda component removed from the EEG data. For reference, we also show the A17 FRP before lambda removal. The shape of the O1-Oz-O2 average waveform was affected only a little by the lambda component removal when compared with Figure 12*A*, resulting in a slightly reduced second peak amplitude. Electrode A17 was affected significantly, resulting in a waveform very similar to the average O1-Oz-O2 potential.

**Figure 26.**
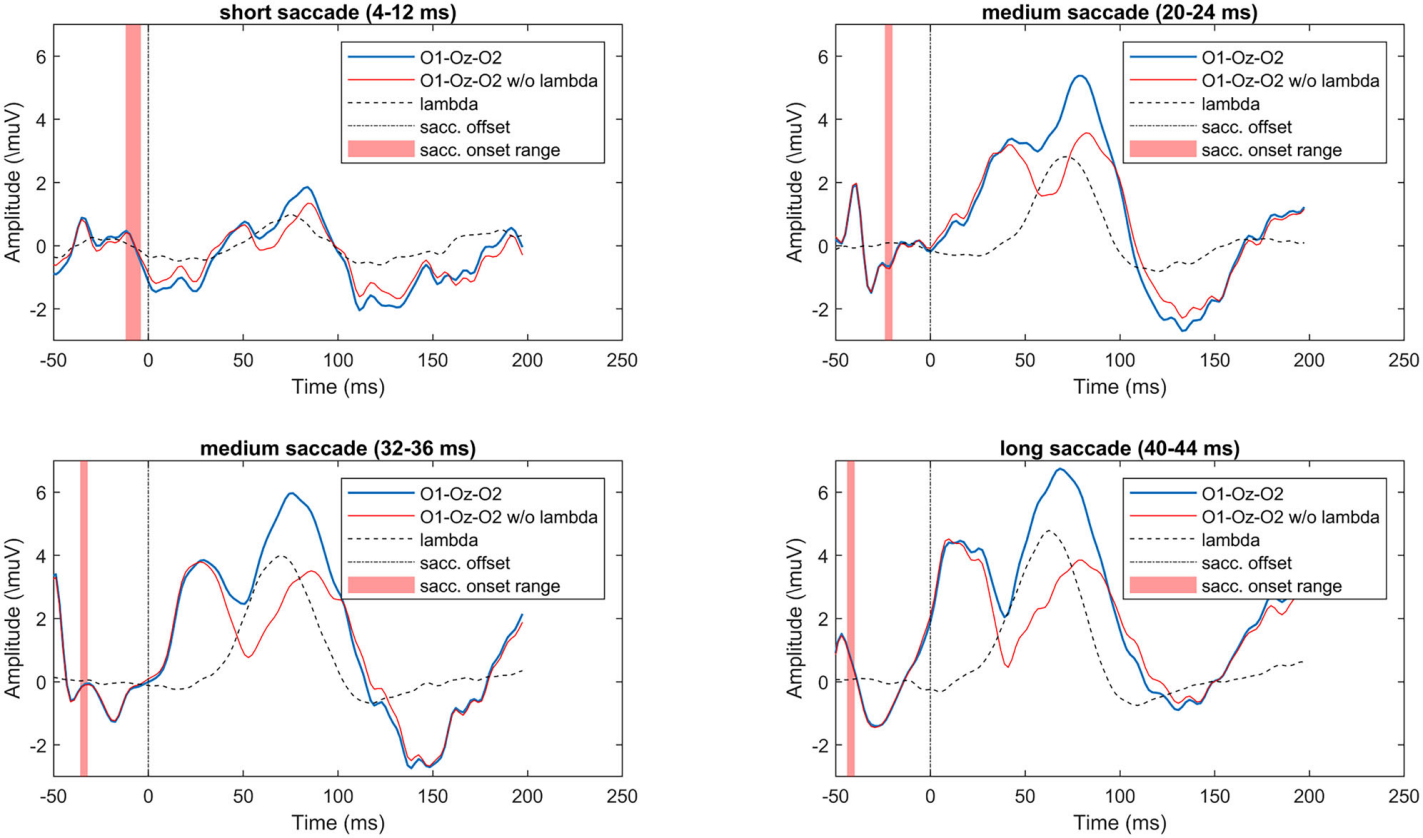
The effect of lambda removal on averaged occipital FRP in case of short, medium, and long saccades. Original occipital FRP in blue, lambda-removed FRP in red. Note that the lambda peak removal affects primarily the second (saccade offset-locked) peak. The first (saccade onset-locked) peak is unaffected.

To further understand the effect of lambda on FRP, we sorted the FRP epochs by saccade duration, and in Figure 26 we plot the occipital FRP waveform before and after removal. Four different saccade durations were used to illustrate the saccade-related temporal effects in the FRP waveform. Each subplot shows averaged waveforms of ∼90 epochs. The FRP consists of two peaks, the first is locked to the saccade onset with an approximate latency of 60 ms, while the second peak is locked to saccade offset with ∼80 ms latency. The removal of the lambda response component affects the second peak; the first peak is mainly unaffected and changes its position with the saccade onset.

**Figure 27.**
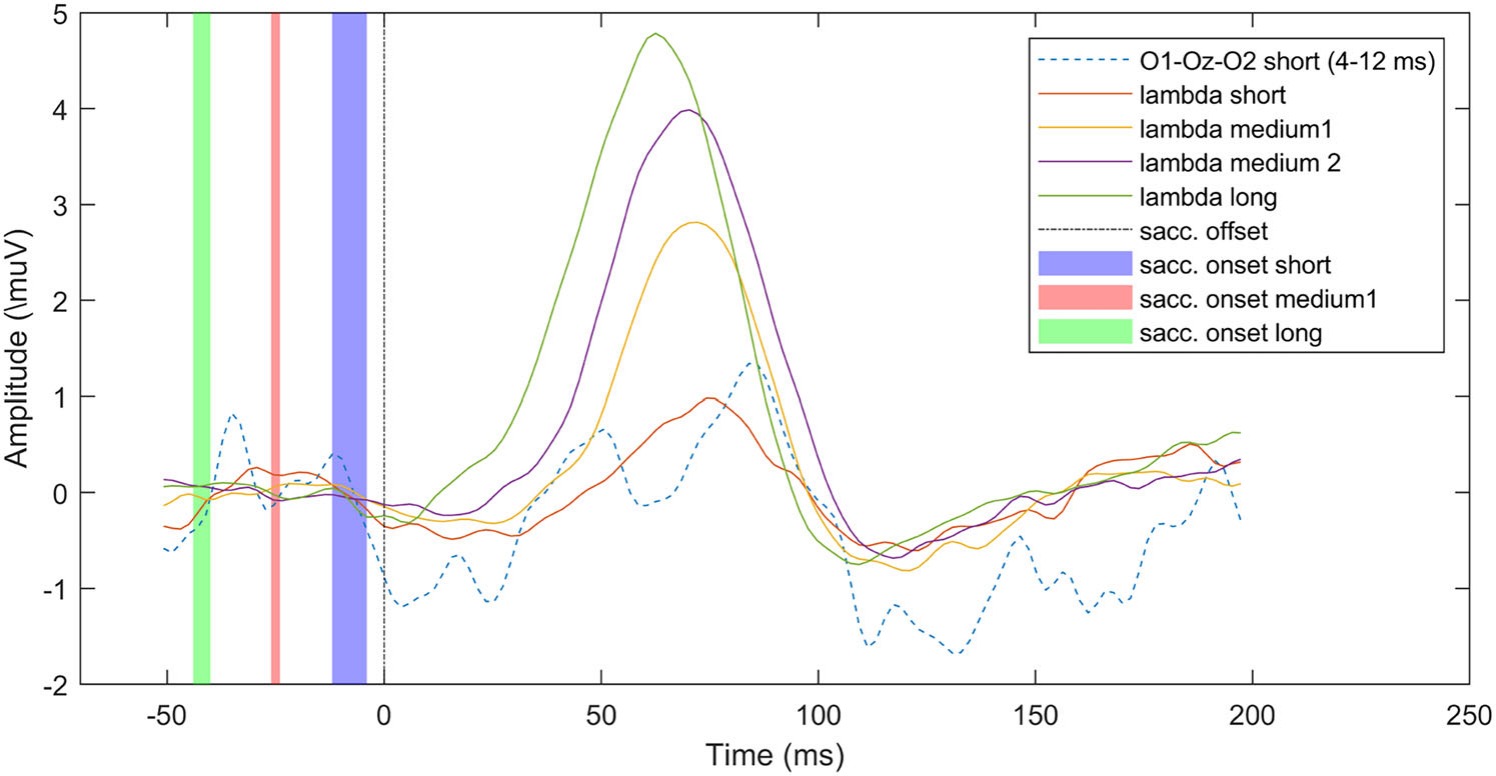
Lambda component modulated by saccade duration.

In Figure 27 we show the effect of saccade duration on the lambda component waveform. As before, we used short, medium, and long saccade groups for illustration. The figure depicts very clearly the modulating effect of saccade length on lambda amplitude. In addition, a subtle temporal shift is detectable; the onset of the lambda waveforms is dependent on the saccade onset. Extended Data Figures 11-1 and 12-1 show ERP images of saccade onset- and offset-locked epochs that provide more details of the occipital processes evoked by saccades.

**Figure 28.**
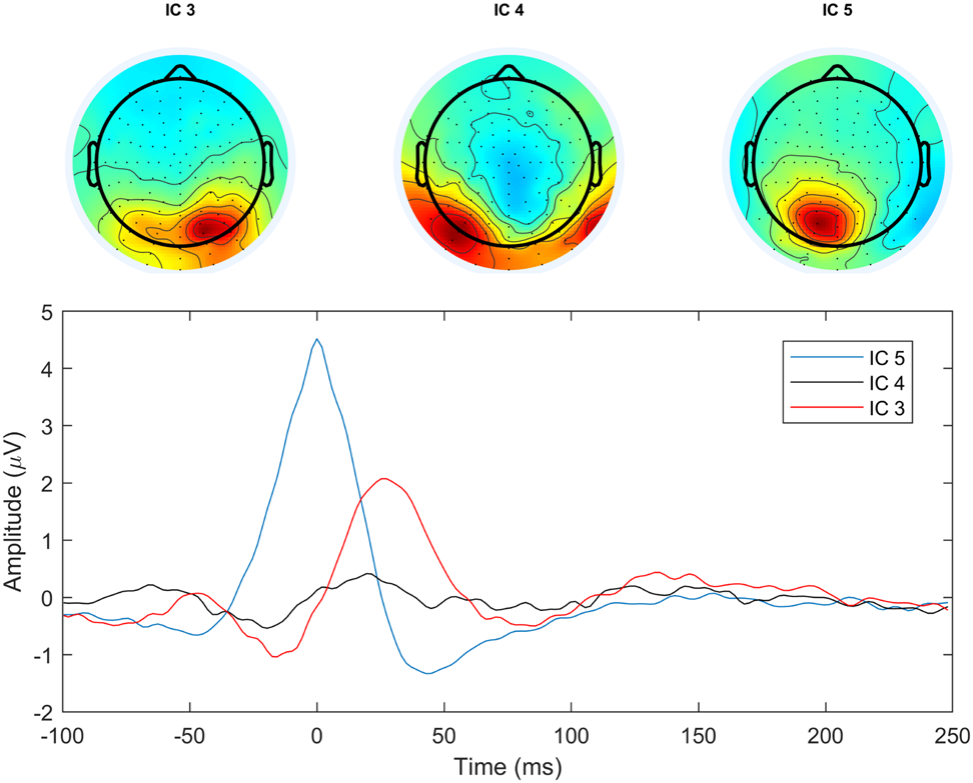
Lambda peak-locked time course of the three separated occipital independent components.

Up to this point we only focused on the lambda component and the occipital FRP. However, the ICA decomposition separated additional occipito-parietal sources, located on the right and left posterior area, as shown in Figure 28. This suggests that the averaged scalp occipital FRP is, indeed, a mixture of several underlying sources.

## Discussion

The current study investigated the use of ICA for separating saccade-related lambda response activity from other neural and non-neural sources activated during art painting viewing. Our goal was to verify whether ICA can uncover a unique lambda response component that later can be used for single-trial analysis. Furthermore, we studied the sensitivity of ICA decomposition to different preprocessing parameters, such as filtering, data length, and sampling rate while extracting the lambda component. For this, we simultaneously recorded eye movements and EEG activity during a free-viewing experiment.

Natural, free-viewing experiments, where participants are encouraged to explore the scenes or their visual environment without restrictions, are intrinsically linked with unpredictable sequences of saccades, fixations, and blinks. Saccades and fixations can now be detected accurately with modern eye-trackers. Their simultaneous use with EEG, however, is not without challenges, especially the identification, characterization, and correction of eye movement artifacts (Dimigen et al., 2011; Plöchl et al., 2012). ICA is commonly used for artifact removal. Dimigen (2020) proposed a sophisticated method to differentiate between various eye-related artifacts (eye balls, eye lids, and extraocular muscles) in free-viewing experiments (Dimigen, 2020). Most reported ICA-based detection methods focused on saccade detection and related artifact removal, affecting only frontal electrodes (Funase et al., 2010; Chaumon et al., 2015).

We are aware of only two studies (Vigon et al., 2002; Miyakoshi et al., 2015) that investigated ICA-based lambda detection, both proving that ICA can extract a lambda component. Vigon et al. (2002) investigated the effect of signal length on the performance of ICA-based lambda extraction in a controlled experiment. They applied ICA to spatially and temporally averaged waveforms and concluded that increasing dataset length by concatenating multiple trials improved the accuracy of ICA for extracting the lambda wave. In the lambda component they found three subcomponents, two positive peaks locked to saccade onset and one positive peak locked to saccade offset as previously described in Thickbroom et al. (1991). Miyakoshi et al. (2015) studied the detection of lambda responses with ICA using fixation onset-locked ERPs obtained from a visual search task based on Gabor patch stimuli. They extracted ∼200 ICs from each of the 20 subjects, then clustered similar components, and identified four posterior IC clusters that construct the scalp-measured lambda response. While our experiment design differed from these studies since we used visual artwork and a free-viewing paradigm, ICA also separated a clear lambda component from other sources. Our findings verified that increasing data length improves lambda extraction quality, but—while the general recommended minimum data points (Onton et al., 2006) for reliable ICA is 20 × channels^2^ (∼10.6 min at fs = 512 Hz)—we were able to detect lambda components even in 4-min-long (*L* = 25%) continuous datasets.

Our results confirm previous findings that high-pass filtering has strong effect on ICA performance (Bigdely-Shamlo et al., 2015; Winkler et al., 2015; Dimigen, 2020). In our case the high-pass filter parameter was the dominating factor in the quality of lambda extraction. At HP = 0.1 Hz there was no parameter combination that could produce a clear lambda component. The hierarchical clustering also confirmed the effect of high-pass filtering, generating four clusters characterized by the three HP cutoff frequency values (C1, 0.5; C2, 0.5; C3, 1; and C4, 2Hz). The first 0.5 Hz cluster contained short (*L* = 25 and 50%) datasets, while the second holding the longer (*L* = 75 and 100%) datasets. The 1 and 2 Hz clusters showed no significant effect to changes in the length parameter. The only case when lambda component was found in a HP = 0.1 Hz filtered dataset was in the synthetic ground truth test, where the signal-to-noise ratio was much better than what is normally found in EEG measurements. Our recommendation is, therefore, that a minimum 1 Hz (preferably 2 Hz) high-pass filter should be used before performing ICA for lambda extraction.

By comparing the shape and timing of the extracted lambda component waveform to saccade onset- and offset-related occipital potentials, we demonstrated that the lambda wave is locked to saccade offset/fixation onset and is highly correlated to the traditionally computed average lambda response (FRP). Our method, however, produced single peak lambda waveforms which are in contrast to the 3-peak lambda component shapes reported by Vigon et al. (2002). The difference is probably due to the spatial averaging they used that reduced the number of electrodes (hence components) considerably, which might have introduced mixing with other occipital processes. As per the number of lambda-related posterior components, we always found only one unique lambda component and one or two additional saccade-related occipital components, as shown in Figure 28. To aid objective and reproducible lambda classification, we proposed an automated procedure that can find the lambda component in the set of independent components produced by ICA. The method proved to be correct and useful in significantly reducing classification time and effort.

The projected scalp location of the lambda activations showed considerable individual variations implying that using a standard electrode or electrode set (e.g., Oz or average of O1-Oz-O2) for computing FRPs in the traditional way may result in distorted waveforms. Using the lambda IC instead of occipital FRP seems to be a more reliable and accurate approach to measure visual response as it automatically eliminates variations in lambda locations produced by cortical anatomy or the placement of electrodes.

Single-trial classification of FRPs and the role of the lambda response in various experimental conditions has become of interest in current research and may enable progress in studying the neural correlates of natural scene perception (Yagi et al., 1998; Brouwer et al., 2013; Touryan et al., 2017; Ries et al., 2018). Yagi (1982) also suggested that lambda responses could serve as objective measures of visual processing. For this, the accurate identification and extraction of individual lambda responses is a crucial step.

The proposed method can be useful in visual perception studies analyzing individual single-trial lambda peaks without signal averaging instead of FRPs that can lead to improved temporal resolution and the extraction of more accurate peak features (amplitude, latency, peak width). It can also be combined with the analysis of other occipito-parietal independent components to understand the correlation between unmixed activation sources during perception.

Our study has some limitations. There were partial saccades that the eye-tracker could not register correctly therefore were not included in the saccade–peak matching process, leaving potential lambda peaks unmatched. In some cases, saccades followed one another quickly producing overlap effects on the lambda peaks. In addition, the peak detection was based on peak prominence. Detection accuracy can be probably improved by using more sophisticated algorithms. Finally, the computational demands of ICA execution (runtime of 6–10 d per subject) limited the extent of our parameter analysis.

In conclusion, we demonstrated that ICA can reliably and uniquely find a lambda independent component that can be used for further single lambda response extraction paving the way for single-trial lambda response analysis in free-viewing, naturalistic settings. With an extensive parameter study, we verified that the detection quality is largely insensitive to preprocessing parameters, except high-pass filtering. Nevertheless, further parameter analyses, benchmarking of ICA, and experimenting with different ICA algorithms for lambda component extraction are recommended.

## Synthesis

Reviewing Editor: Niko Busch, Westfalische Wilhelms-Universitat Munster Decisions are customarily a result of the Reviewing Editor and the peer reviewers coming together and discussing their recommendations until a consensus is reached. When revisions are invited, a fact-based synthesis statement explaining their decision and outlining what is needed to prepare a revision will be listed below. The following reviewer(s) agreed to reveal their identity: Makoto Miyakoshi.

# Synthesis

Thank you for submitting the revised version of your manuscript. One of the original reviewers recommends acceptance, while the other was unfortunately not available to re-review the manuscript.

I have carefully evaluated the revision and your responses to both reviewers. I concur that the revision is well done and that the reviewer comments have been sufficiently addressed.

I am in principle in favor of accepting the paper in its present form. My decision for "Revise - Reviewing Editor Review Only" is just to give you the opportunity to respond to the reviewer's remaining comment regarding ICA polarity ambiguity. In line with the reviewer's note, I leave it open whether you wish to incorporate changes into the manuscript or simply take the comment as "food for thought."

Best regards,

Niko Busch

# Reviewer 1

The paper will add another successful application of computational neuroscience in human cognitive science.

The authors have thoroughly addressed all of my previous comments. The revised manuscript is substantially improved and scientifically sound. I have no further concerns and recommend it for publication in its current form.

One final note: the authors might find the following paper useful when discussing ICA's polarity indeterminacy, as raised by Reviewer 2. I am not suggesting that they apply this method to the current data, since doing so might alter the results (though likely not the conclusions).

Nakanishi, M., &Miyakoshi, M. (2023). Revisiting Polarity Indeterminacy of ICA-Decomposed ERPs and Scalp Topographies. Brain Topography, 36, 223-229. https://doi.org/10.1007/s10548-023-00944-1 (PMID: 36840814)

ICA's polarity indeterminacy has often been regarded as an unwelcome mathematical artifact. However, this paper discusses potential missed opportunities due to waveform cancellation caused by naïve averaging across polarity-reversed ERPs. It then proposes a solution using a generalized eigenvalue decomposition framework to optimize ERP polarities across ICs.
