# Supplementary figures and images for: Reliable Single-Trial Detection of Saccade-Related Lambda Responses with Independent Component Analysis
Source: eNeuro. 2025 Nov 21;12(11):ENEURO.0270-25.2025. doi: 10.1523/ENEURO.0270-25.2025 (PMC12657691; doi:10.1523/ENEURO.0270-25.2025)

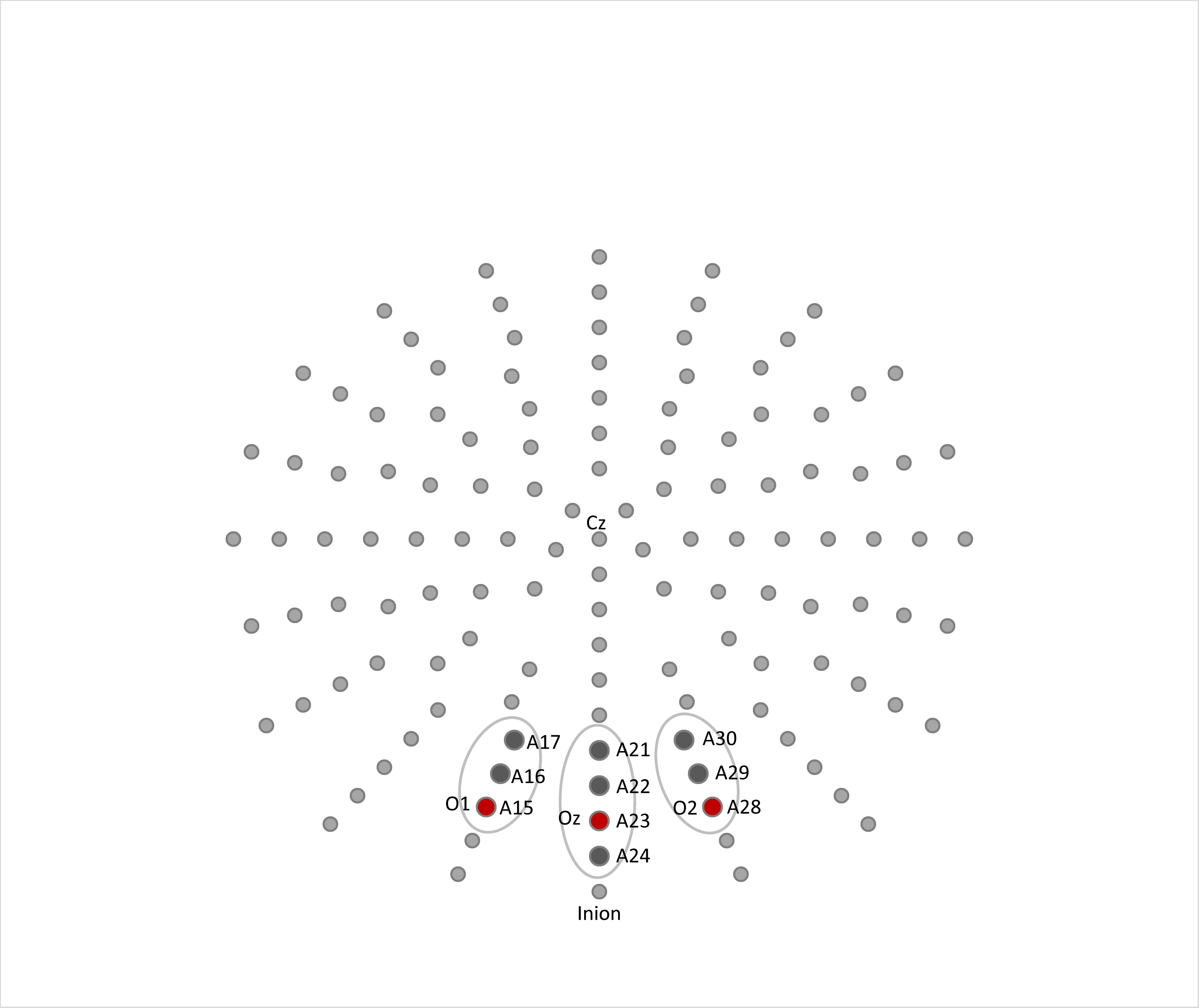

Supplement: Figure 4-1 — Biosemi 128-channel ABC layout with O1, Oz, O2 and occipito-parietal electrodes highlighted. Download Figure 4-1, TIF file. [file eneuro-12-ENEURO.0270-25.2025-s005.tif]

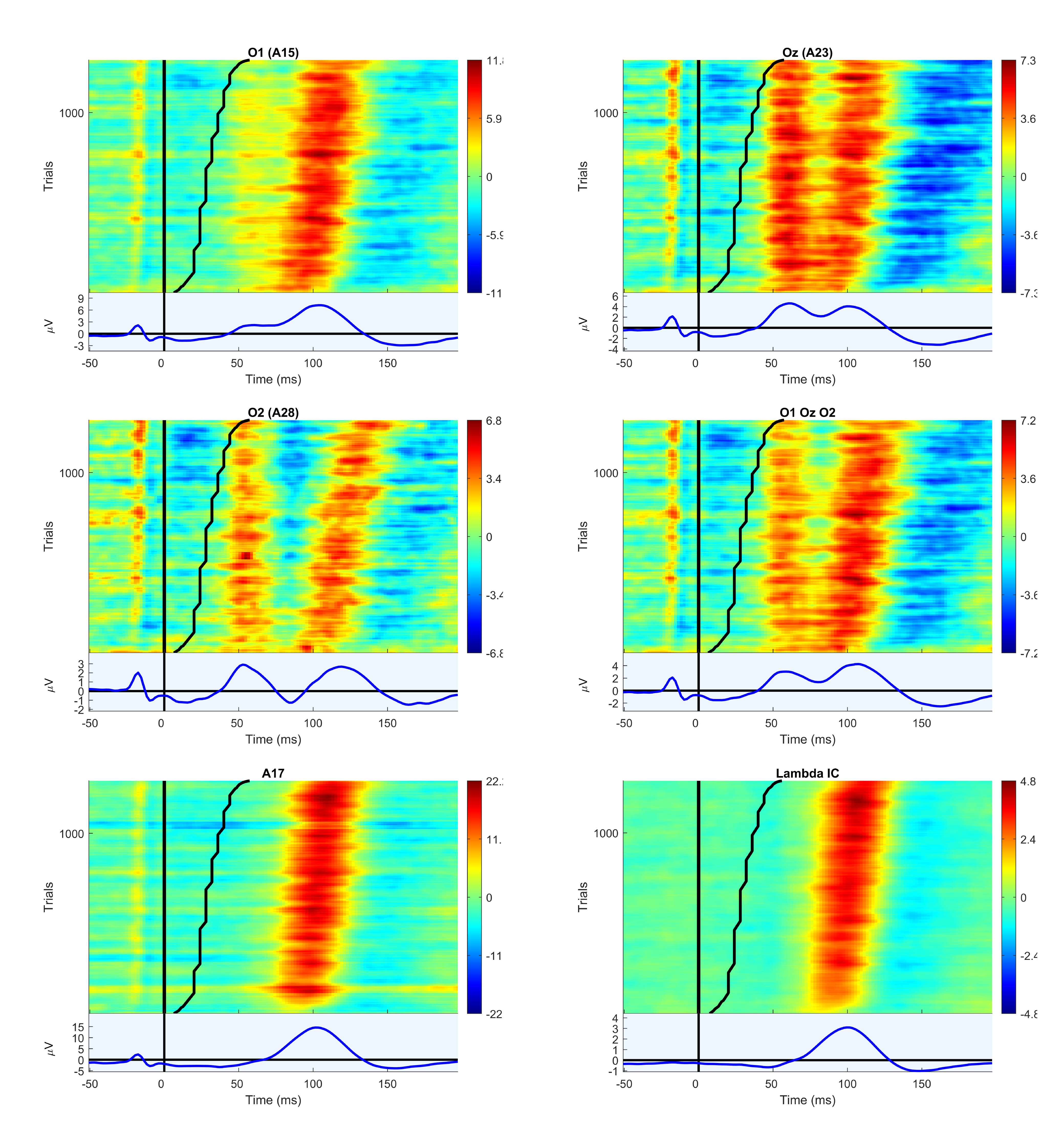

Supplement: Figure 11-1 — Saccade onset locked ERP images of channel O1 (A15), Oz (A23), O2 (A28), average of O1-Oz-O2 and A17 vs. IC5 (lambda independent component) epochs sorted by saccade duration measured on participant S7. Other participants show similar results. The occipital ERPs show the saccadic spike potential SP (-20 ms) and two distinct peaks with varying amplitudes. The first peak follows saccade onset by ∼ 50 ms, the second peak follows saccade offset by ∼100 ms. The average ERP waveform (blue plot at the bottom of each pane) cannot depict the temporal jitter hence the behavior of the offset-locked peak latencies. Download Figure 11-1, TIF file. [file eneuro-12-ENEURO.0270-25.2025-s004.tif]

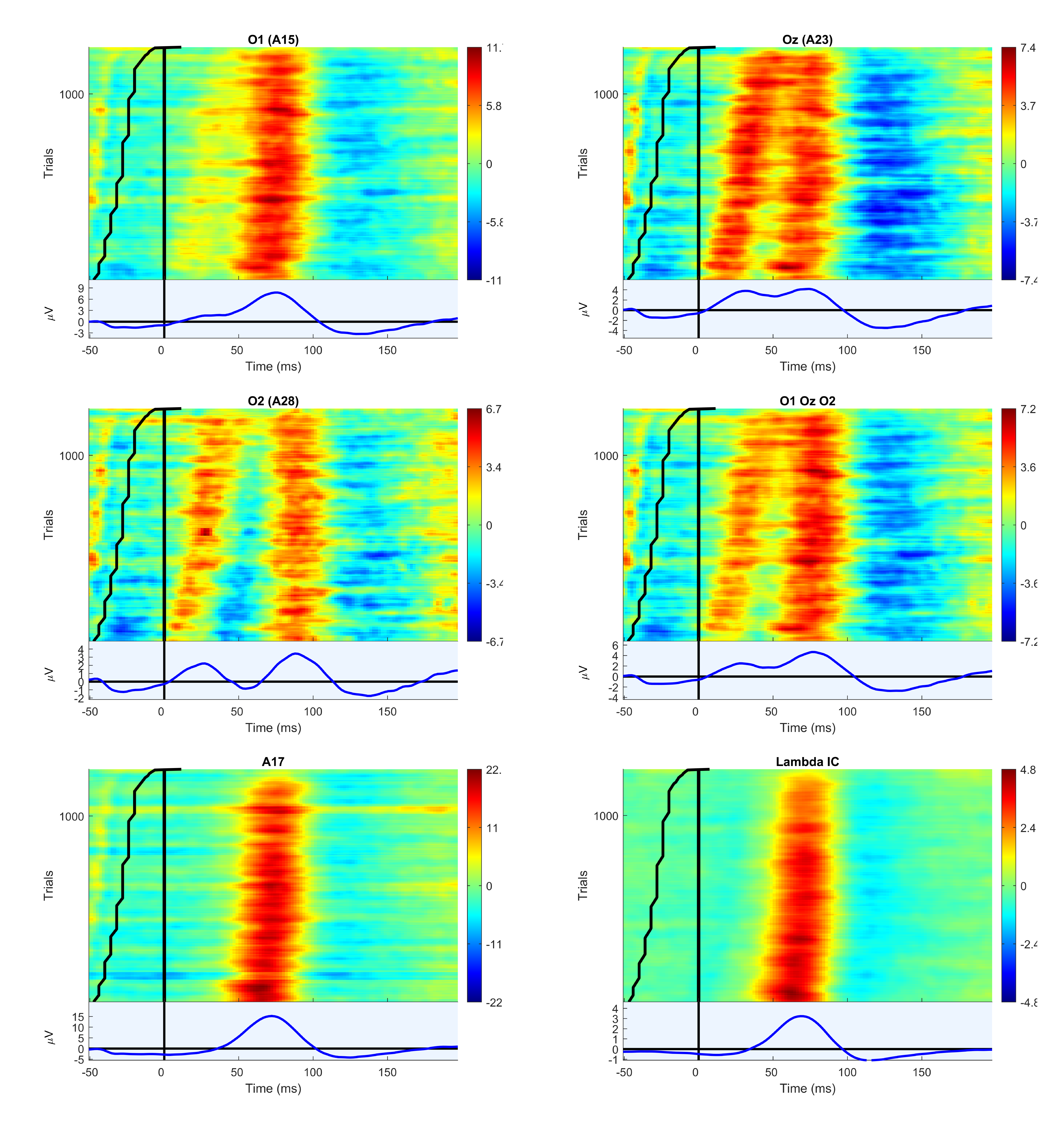

Supplement: Figure 12-1 — Saccade offset locked ERP images of channel O1 (A15), Oz (A23), O2 (A28), average of O1-Oz-O2 and A17 vs. IC5 (lambda independent component) epochs sorted by saccade duration measured on participant S7. The second (offset locked) peak shows saccade onset influence; the onset of longer saccades moves the location of the offset-locked second peak slightly forward in time (most prominent at O1 and Oz). Download Figure 12-1, TIF file. [file eneuro-12-ENEURO.0270-25.2025-s003.tif]

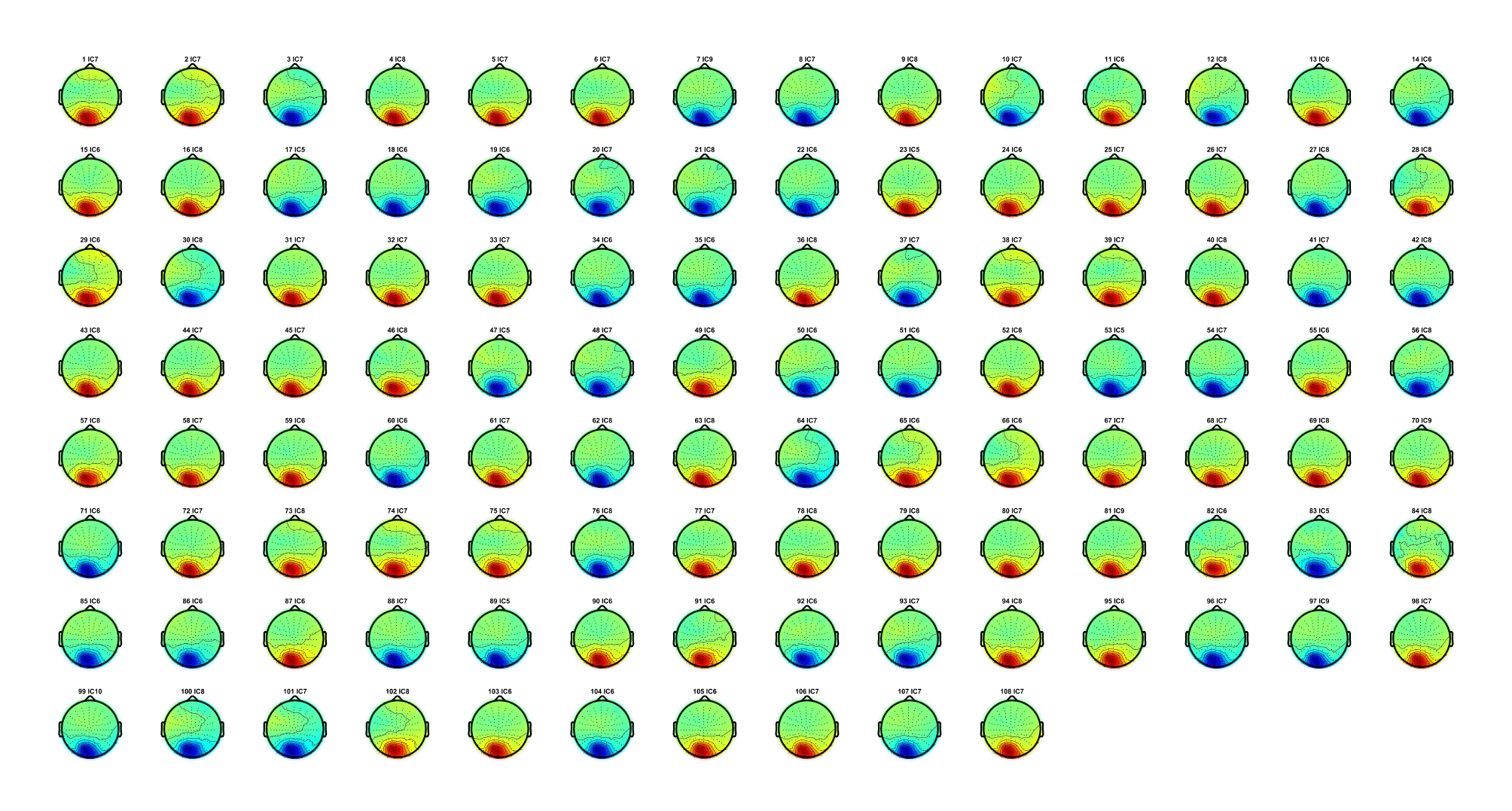

Supplement: Figure 22-1 — Projected scalp maps of the extracted component after performing automatic lambda identification on the 108 HP {greater than or equal to} 0.5 Hz parameter combination pre-processed datasets. Download Figure 22-1, TIF file. [file eneuro-12-ENEURO.0270-25.2025-s002.tif]
